# Upregulation of galectin-3 in influenza A virus infection promotes viral RNA synthesis through its association with viral PA protein

**DOI:** 10.1186/s12929-023-00901-x

**Published:** 2023-02-23

**Authors:** Mei-Lin Yang, Yi-Cheng Chen, Chung-Teng Wang, Hao-Earn Chong, Nai-Hui Chung, Chia-Hsing Leu, Fu-Tong Liu, Michael M. C. Lai, Pin Ling, Chao-Liang Wu, Ai-Li Shiau

**Affiliations:** 1 Department of Microbiology and Immunology, College of Medicine, National Cheng Kung University, 1, University Road, Tainan, 701401 Taiwan; 2 Ditmanson Medical Foundation Chia-Yi Christian Hospital, Chiayi, Taiwan; 3 Department of Biochemistry and Molecular Biology, College of Medicine, National Cheng Kung University, 1, University Road, Tainan, 701401 Taiwan; 4 Institute of Biomedical Sciences, Academia Sinica, Taipei, Taiwan; 5 Graduate Institute of Biomedical Sciences, China Medical University, Taichung, Taiwan; 6 Institute of Molecular Biology, Academia Sinica, Taipei, Taiwan

**Keywords:** Galectin-3, Influenza virus, vRNP import, RNA-dependent RNA polymerase, RNA synthesis, Viral PA

## Abstract

**Background:**

Influenza is one of the most important viral infections globally. Viral RNA-dependent RNA polymerase (RdRp) consists of the PA, PB1, and PB2 subunits, and the amino acid residues of each subunit are highly conserved among influenza A virus (IAV) strains. Due to the high mutation rate and emergence of drug resistance, new antiviral strategies are needed. Host cell factors are involved in the transcription and replication of influenza virus. Here, we investigated the role of galectin-3, a member of the β-galactoside-binding animal lectin family, in the life cycle of IAV infection in vitro and in mice.

**Methods:**

We used galectin-3 knockout and wild-type mice and cells to study the intracellular role of galectin-3 in influenza pathogenesis. Body weight and survival time of IAV-infected mice were analyzed, and viral production in mouse macrophages and lung fibroblasts was examined. Overexpression and knockdown of galectin-3 in A549 human lung epithelial cells were exploited to assess viral entry, viral ribonucleoprotein (vRNP) import/export, transcription, replication, virion production, as well as interactions between galectin-3 and viral proteins by immunoblotting, immunofluorescence, co-immunoprecipitation, RT-qPCR, minireplicon, and plaque assays. We also employed recombinant galectin-3 proteins to identify specific step(s) of the viral life cycle that was affected by exogenously added galectin-3 in A549 cells.

**Results:**

Galectin-3 levels were increased in the bronchoalveolar lavage fluid and lungs of IAV-infected mice. There was a positive correlation between galectin-3 levels and viral loads. Notably, galectin-3 knockout mice were resistant to IAV infection. Knockdown of galectin-3 significantly reduced the production of viral proteins and virions in A549 cells. While intracellular galectin-3 did not affect viral entry, it increased vRNP nuclear import, RdRp activity, and viral transcription and replication, which were associated with the interaction of galectin-3 with viral PA subunit. Galectin-3 enhanced the interaction between viral PA and PB1 proteins. Moreover, exogenously added recombinant galectin-3 proteins also enhanced viral adsorption and promoted IAV infection in A549 cells.

**Conclusion:**

We demonstrate that galectin-3 enhances viral infection through increases in vRNP nuclear import and RdRp activity, thereby facilitating viral transcription and replication. Our findings also identify galectin-3 as a potential therapeutic target for influenza.

**Supplementary Information:**

The online version contains supplementary material available at 10.1186/s12929-023-00901-x.

## Background

Influenza is one of the most prevalent and significant viral infections worldwide. Influenza viruses are members of the *Orthomyxoviridae* and classified into types A, B, and C based on antigenic differences in their nucleoprotein (NP) and matrix protein 1 (M1). Influenza A virus (IAV) is further subtyped based on the antigenicity of the two surface glycoproteins hemagglutinin (HA) and neuraminidase (NA). Vaccines and antiviral drugs are required to combat influenza. However, high mutation rates of IAV facilitate the emergence of viral escape mutants, rendering vaccines and antiviral drugs directly against virus-encoded targets potentially ineffective [1]. Therefore, identification of new therapeutic targets is urgently needed. Since interactions between viruses and host factors are crucial for viral entry, viral replication, assembly, and trafficking, understanding of the interplay between IAV and host factors may provide new targets for antiviral therapy [2, 3].

The genome of influenza virus contains eight segments of negative-sense single-stranded RNA that form viral ribonucleoprotein (vRNP) complexes by association of the NP with trimeric RNA-dependent RNA polymerase (RdRp) that consists of the polymerase acidic protein (PA), polymerase basic protein 1 (PB1), and polymerase basic protein 2 (PB2). The steps of the viral life cycle include binding of the HA to sialic acid receptors on the host cell surface, internalization of the virion into the endosome, and release of the vRNP complex into the cytoplasm triggered by low pH via the viral matrix protein 2 (M2) ion channel protein [4]. Then, the vRNP is transported into the nucleus, which is facilitated by the viral NP, and the transcription and replication are initiated by viral RdRp. Viral RNA synthesis occurs in the nucleus and is divided into two stages. First, viral RNA (vRNA) transcribes to mRNA through a unique process by the PA-dependent “cap-snatching” reaction on cellular capped RNAs to generate short capped RNA primers. Then, the PB2 subunit binds the capped RNA primer, and viral mRNA synthesis is performed by the PB1 subunit. After newly viral proteins are translated, replication of vRNA proceeds via complementary RNA (cRNA) intermediates and inverses back to vRNA in a primer-independent manner. The virus uses host cell factors to help these processes. Several cellular factors involved in cell transcription, RNA processing, or RNA binding have been identified as positive or negative regulators for viral transcription and replication by interacting with components of the RdRp or the NP [5–8]. In the final stage of the viral life cycle, progeny vRNP and other viral proteins assemble into the virion particles, which are then released from the cell surface.

Galectin-3, a member of the β-galactoside-binding protein family, has diverse intracellular and extracellular roles in physiological and pathophysiological conditions, such as cell growth, cell adhesion, cell–cell interactions, apoptosis, angiogenesis, and mRNA processing [9]. Galectin-3 can be detected intracellularly in transport vesicles, the cytoplasm, and the nucleus, as well as in the extracellular milieu [10]. Galectins may serve as a pathogen-recognition receptor for a variety of viruses and bacteria. We have previously shown that galectin-1 directly binds to the surface of intact influenza virions and inhibits viral infection in vitro and in vivo [11]. Regarding galectin-3, intracellular galectin-3 can promote HIV-1 budding through enhancing the interaction of ALG-2-interacting protein X (Alix) with viral Gag p6 in T cells [12]. Galectin-3 is required for efficient uptake and infection of minute virus of mice, and viral infectivity is positively correlated with galectin-3 expression in human cancer cell lines [13]. Galectin-3 has also been identified as a herpes simplex virus type 1 entry mediator in corneal keratinocytes, thus facilitating viral infection [14]. Furthermore, a study with a mouse model of pneumococcal pneumonia after influenza has shown that galectin-1 and, in particular, galectin-3 expressed and secreted in the airway epithelial cells upon IAV infection bind strongly to IAV and *Streptococcus pneumoniae* [15]. Moreover, NA of IAV or *S. pneumoniae* can desialylate airway epithelial cells, resulting in enhancing pneumococcal adhesion through galectin binding. These results suggest that galectin-1 and galectin-3 may contribute to pneumococcal pneumonia after influenza infection. By promoting the activation of the nucleotide oligomerization domain-like receptor protein 3 (NLRP3) inflammasome [16], intracellular galectin-3 enhances lung inflammation in mice infected with avian H5N1 IAV [17]. Contrary to the enhancement of viral infection, galectin-3 has been shown to inhibit or interfere with viral infection. Intracellular galectin-3 can inhibit the replication of porcine reproductive and respiratory syndrome virus by interacting with viral Nsp12 protein in vitro [18]. Moreover, administration of recombinant galectin-3 proteins can inhibit IAV replication in cell culture [19]. Since galectins may exert differential effects depending on whether they act intracellularly or extracellularly [20], the pathophysiological role of galectin-3 in IAV infection remains unclear.

In the present study, we investigated the impact of galectin-3 on IAV infection in cell culture and mice. Furthermore, we dissected which step(s) of the viral life cycle was affected by galectin-3. We demonstrate that galectin-3 interacts with the PA subunit of the RdRp and promotes the interaction between PA and PB1 proteins, thereby enhancing the RdRp activity. Given the crucial role of the viral PA in the virus life cycle and vial PA–PB1 interaction as an attractive drug target [21, 22], influenza virus takes advantages of host galectin-3 for their efficient replication. Our results also suggest that inhibition of galectin-3 may be a potential therapeutic strategy for influenza.

## Materials and methods

### Mice, cells, influenza viruses, and reagents

Galectin-3 knockout (Gal-3^−/−^) mice with the C57BL/6 background have been described [23]. C57BL/6 mice were purchased from the Laboratory Animal Center of National Cheng Kung University (NCKU) or National Laboratory Animal Center (Taipei, Taiwan). All animal work was carried out using 5- to 6-week-old female mice in animal biosafety level 2 facilities at NCKU. Human A549 lung epithelial, canine MDCK kidney, and murine RAW 264.7 macrophage cell lines were purchased from the Bioresource Collection and Research Center (Hsinchu, Taiwan). Human 293T embryonic kidney cells were obtained from National RNAi Core Facility, Academia Sinica, Taiwan. Unless stated otherwise, all cells were cultured in Dulbecco’s modified Eagle’s medium (DMEM) supplemented with 10% cosmic calf serum (Hyclone, Logan, UT, USA), 2 mM L-glutamine, and 50 µg/ml gentamicin. A549 cells were cultured in F12 medium containing 10% fetal bovine serum (Hyclone), 2 mM L-glutamine, and 50 µg/ml gentamicin. Primary murine lung fibroblasts from Gal-3^−/−^ and wild-type (WT) mice were isolated by trypsin digestion of minced lung tissue and used between the fourth and seventh passages as previously described [24]. Bone marrow cells isolated from Gal-3^−/−^ and WT mice were cultured in RPMI 1640 medium containing 10% fetal bovine serum and treated with 50 ng/ml of macrophage colony-stimulating factor (416-ML-010, R&D, Minneapolis, MN, USA) for 7 days to generate bone marrow-derived macrophages (BMDMs) [25]. Influenza A/WSN/33 (H1N1) viruses originally obtained from National Institute of Allergy and Infectious Diseases (Bethesda, MD, USA) were propagated and titrated in MDCK cells and used in all experiment unless otherwise stated [11]. Influenza A/PR/8/34 (H1N1) viruses were generated with the eight-plasmid reverse genetics system [25, 26]. All in vitro work on influenza viruses was carried out in biosafety level 2 laboratories. Human recombinant galectin-3 proteins and bafilomycin A1 were purchased from ProteinTech (Chicago, IL, USA) and Cayman Chemical Company (Ann Arbor, MI, USA), respectively. Cycloheximide and TPCK-trypsin were obtained from SigmaAldrich (St. Louis, MO, USA).

### Construction of human galectin-3 eukaryotic expression vector and mouse galectin-3 prokaryotic expression vector and production of recombinant galectin-3 proteins

To overexpress human galectin-3 in A549 cells, we constructed a Flag-tagged galectin-3 expression plasmid driven by the CMV promoter. Briefly, the coding region of human galectin-3 was amplified from the cDNA of A549 cells by polymerase chain reaction (PCR) amplification with sense primer 5′-TCCAAGCTTATGGCAGACAATTTTTCG-3′ and antisense primer 5′-TCCCTCGAGTATCATGGTATATGAAGCAC-3′, in which *Hin*dIII (underlined) and *Xho*I (underlined) sites were introduced onto the 5′- and 3′-ends, respectively. The resulting PCR product was digested with *Hin*dIII and *Xho*I, and cloned into pCR3.1-Flag plasmid at the *Hin*dIII/*Xho*I sites to generate pCR3.1-Gal-3-Flag. To construct a prokaryotic expression vector encoding mouse galectin-3, the coding region of mouse galectin-3 was amplified from the cDNA of DC2.4 cells, a murine dendritic cell line, by PCR amplification with sense primer 5′-ATGGCAGACAGCTTTTCGCTT-3′ and antisense primer 5′-TTAGATCATGGCGTGGTTAGCG-3′. The resulting PCR product was ligated to the TA cloning vector yT&A (Yeastern Biotech, Taipei, Taiwan), digested with *Hin*dIII, and subcloned into the pRSET-C bacterial expression vector (Invitrogen, Carlsbad, CA, USA) at the *Hin*dIII sites to generate pRSET-C-mGal-3. Galectin-1 prokaryotic expression vector has been described previously [11]. Histidine-tagged galectin-3 and galectin-1 proteins were prepared as previously described [11].

### Generation of lentiviral vectors encoding small hairpin RNA (shRNA)

The pLKO.1-puro-based lentiviral vectors encoding shRNAs for human galectin-3, including TRCN0000029304 (designated shgal-3 #4), TRCN0000029305 (designated shgal-3 #5), TRCN0000029307 (designated shgal-3 #7), and TRCN0000029308 (designated shgal-3 #8), and for β-galactosidase (TRCN0000072240, designated shLacZ) were obtained from National RNAi Core Facility, Academia Sinica, Taiwan. The sequence of shgal-3 #4 targets to nucleotides of human galectin-3 (Genbank: NM_002306) from 699 to 719, shgal-3 #5 targets to nucleotides from 533 to 553, shgal-3 #7 targets to nucleotides from 710 to 730, and shgal-3 #8 targets to nucleotides from 571 to 591. All target sequences of galectin-3 shRNAs are galectin-3-specific and in the carbohydrate recognition domain (CRD) of human galectin-3. Recombinant lentiviruses encoding shRNAs specific to human galectin-3 and β-galactosidase were individually produced by transient transfection of 293T cells with various pLKO.1-puro shRNA plasmids along with the packaging plasmid psPAX2 and the VSV-G expression plasmid pMD2G [27]. The knockdown efficiency of galectin-3 shRNA was confirmed by immunoblot analysis. All work on recombinant lentiviral vectors was conducted in biosafety level 2 laboratories.

### Animal studies

Groups of Gal-3^−/−^ and C57BL/6 mice were intranasally inoculated with 10^5^ plaque-forming units (PFU) of IAV which corresponded to 1.5 × median lethal dose (LD_50_) at day 0. The mice were monitored daily for illness and death for 14 days after viral infection. To detect galectin-3 in the bronchoalveolar lavage (BAL) fluid of mice infected with IAV, C57BL/6 mice were intranasally or intratracheally inoculated with 10^5^ PFU of IAV. At different time points, the BAL fluid was collected by injection of 1 ml of sterile saline to the alveolar space of mice through the trachea and immediate aspiration by gentle suction, and cell-free supernatant was obtained and frozen at − 70 °C as previously described [11]. In addition, the lungs of mice were removed, formalin-fixed, and paraffin-embedded for further use. To test the effect of recombinant mouse galectin-3 proteins on IAV-infected mice, C57BL/6 mice were intratracheally inoculated with 10^6^ PFU of IAV at day 0 and treated with recombinant mouse galectin-3 proteins (50 µg) or bovine serum albumin (BSA) at days 2, 4, and 5 via the same route. The mice were monitored daily for illness and death after viral infection.

### Immunoblotting, immunohistochemistry, co-immunoprecipitation, and enzyme-linked immunosorbent assay (ELISA)

Immunoblot analysis was performed to detect galectin-3 and influenza virus proteins in mouse lung tissue, BMDMs, as well as A549 and 293T cells using standard methods. The primary antibodies used for immunoblotting included monoclonal antibodies specific for the NP of all influenza A strains (ab20343, 1:3000; Abcam, Cambridge, UK), mouse monoclonal anti-influenza A NS1 antibody (sc-130568, 1:1000, Santa Cruz Biotechnology, Santa Cruz, CA, USA), mouse monoclonal anti-influenza A matrix protein antibody (MCA401, 1:1000, AbD Serotec, Oxford, UK), rabbit anti-human galectin-3 antibody (sc-20,157, 1:1000, Santa Cruz), rabbit anti-influenza A PA antibody (GTX125932, 1:1000, GeneTex, Irvine, CA, USA), rabbit anti-Flag antibody (20543-1-AP, 1:2000, Proteintech), rabbit anti-HA probe antibody (sc-805, 1:1000, Santa Cruz), and mouse monoclonal anti-β-actin peroxidase antibody (A3854, 1:10000, Sigma-Aldrich). Horseradish peroxidase (HRP)-conjugated goat anti-mouse IgG (115-035-003, 1:4000, Jackson Immuno Research, West Grove, PA, USA) and goat anti-rabbit IgG (111-035-003, 1:4000, Jackson Immuno Research) were used as secondary antibodies. For immunohistochemical staining, tissue sections of formalin-fixed, paraffin-embedded mouse lungs were deparaffinized and antigen-retrieved for examining galectin-3 expression as previously described [24]. We used rabbit anti-human galectin-3 antibody (sc-20157, 1:200, Santa Cruz) as the primary antibody, HRP-conjugated goat anti-rabbit IgG (111-035-003, 1:200, Jackson Immuno Research) as the secondary antibody, 3-amino-9-ethylcarbazole (AEC) as the chromogen, and hematoxylin for the counterstain.

For the co-immunoprecipitation assay, plasmids expressing HA-tagged viral proteins or the Myc-tagged viral PA protein were used as described previously [6, 28, 29]. Briefly, 293T cells were cotransfected with one of the plasmids expressing viral proteins and pCR3.1-Gal-3-Flag, or cotransfected with p3xMyc-PA, pCAG-PB1-HA, and pCR3.1-Gal-3-Flag for 48 h. The cell lysates were incubated with the anti-HA affinity matrix (A2095, Sigma-Aldrich) and extensively washed three times with the wash buffer [50 mM Tris (pH 7.5), 150 mM NaCl, 1% Triton X-100, and 0.5% SDS]. The immunoprecipitated proteins were subjected to immunoblot analysis. Protein-antibody complexes were detected by the ECL system (Millipore, Bedford, MA, USA) and visualized with the BioSpectrum imaging system (UVP Inc., Upland, CA, USA). Relative intensities of protein bands were quantified using the public-domain image analysis package ImageJ software from National Institutes of Health (Bethesda, MD, USA). To detect galectin-3 in culture supernatants of A549 cells and their derivatives, cells (3 × 10^5^) were cultured with 1 ml of the culture medium in 12-well plates for 48 h, and their conditioned medium was collected for quantifying galectin-3 levels using a DuoSet ELISA kit (DY1154, R&D).

### Immunofluorescence and confocal microscopy

For indirect immunofluorescence staining, cells were fixed with 4% formaldehyde for 15 min, permeabilized with 0.1% Triton X-100 for 15 min, blocked with 1% BSA for 60 min, and then probed with the indicated primary antibodies, including mouse monoclonal anti-IAV NP antibody (1331, 1:200, ViroStat, Portland, ME, USA), rabbit anti-human galectin-3 antibody (sc-20157, 1:200, Santa Cruz), rabbit anti-influenza A PA antibody (GTX125932, 1:500, GeneTex), and mouse monoclonal anti-Flag M2 antibody (F1804, 1:500, Sigma-Aldrich), overnight at 4 °C. Alexa Flour 488-goat anti-mouse IgG (A-21202, 1:200, Invitrogen) and Alexa Flour 594-goat anti-rabbit IgG (A-11012, 1:200, Invitrogen) were used as secondary antibodies. The nuclei were counterstained with DAPI (Sigma-Aldrich). Images were acquired using an Olympus FV1000 confocal microscope or observed under fluorescence microscopy (Olympus, Tokyo, Japan).

### IAV binding and internalization assays

For the binding assay, A549 cells transduced with the lentiviral vector encoding shgal-3 #4, shgal-3 #7, or shLacZ were infected with IAV at a multiplicity of infection (MOI) of 5 at 4 °C for 60 min and then washed twice with ice-cold phosphate-buffered saline (PBS) to remove unbound viruses. For the internalization assay, the same transduced cells were infected with IAV at an MOI of 5 at 37 °C for 40 min and then washed twice with acid PBS (pH 1.3) at 4 °C to remove uninternalized viruses. Subsequently, the cell lysates were subjected to immunoblot analysis.

### Analysis of nuclear import of the vRNP complex

To analyze vRNP import into the nucleus, A549 cells that had been transduced with the lentiviral vector encoding shgal-3 #4, shgal-3 #7, or shLacZ were infected with IAV at an MOI of 100 at 37 °C for 60 min and then washed twice with PBS to remove unbound viruses. Subsequently, cells were incubated with 100 µg/ml of cycloheximide for an additional 2 h and fixed with 4% formaldehyde for further immunofluorescence staining. Infected cells treated with 100 nM of bafilomycin A1 served as a positive control for inhibiting vRNP nuclear import [30].

### Reverse transcription quantitative real-time polymerase chain reaction (RT-qPCR)

Total cellular RNA was extracted using the High Pure RNA Isolation Kit (Roche Diagnostics GmbH, Mannheim, Germany) according to the manufacturer’s protocols. A REVERSE-IT first-strand synthesis kit (Applied Biosystems, Foster, CA, USA) was used for cDNA synthesis and qPCR was carried out with QuantiNova SYBR Green PCR kit (Qiagen, Hilden, Germany) on the Prism 7000 sequence detection system (Applied Biosystems). GAPDH served as an internal control for normalization of cellular RNA and intracellular viral RNA. The following primers were used for detecting IAV NP vRNA [31]: sense 5′-GGCCGTCATGGTGGCGAAT-3′ and antisense 5′-CTCAATATGAGTGCAGACCGTGCT-3′; for IAV NP cRNA: sense 5′- GCTAGCTTCAGCTAGGCATC-3′ and antisense 5′-CGATCGTGCCCTCCTTTG-3′; and for IAV NP mRNA: sense 5´-CCAGATCGTTCGAGTCGT-3´ and antisense 5´- CGATCGTGCCCTCCTTTG-3. The primers for detecting GAPDH were sense 5′-AGCCACATCGCTCAGACAC-3′ and antisense 5′-GCCCAATACGACCAAATCC-3′.

### Minireplicon assay

293T cells were cotransfected with the firefly luciferase reporter plasmid pPolI-Luc under the control of the RNA polymerase I promoter and four plasmids for expression of the IAV proteins PA, PB1, PB2, and NP [5]. The total protein amount was used as an internal control to normalize the transfection efficiency. At 48 h post-transfection, cell lysates were measured for luciferase activities with D-luciferin (Synchem, Felsberg, Germany) as the substrate using a luminometer (Lumat LB 9507, Berthold Technologies, Bad Wildbad, Germany).

### Time-of-addition assay

The time-of-addition experiment was performed as previously described with modifications [32]. A549 cells (4.2 × 10^5^) cultured in 6-well plates overnight were infected with IAV at an MOI of 1 at 37 °C for 1 h (time − 1 to 0 h). The cells were then washed with PBS for three times and replenished with fresh serum-free DMEM containing 1 µg/ml of TPCK-trypsin at 37 °C. Human recombinant galectin-3 proteins (0.12, 0.25, 0.5, 1, and 2 µg/ml) was present from − 2 to 8 h (i.e. 1 h before infection, during infection, and after infection for 8 h). To explore the effect of galectin-3 on different steps of viral infection, galectin-3 proteins were added at six time points (− 1 to − 2, − 1 to 0, 0 to 2, 2 to 4, 4 to 6, and 6 to 8 h). After each treatment, the cells were washed with PBS for three times and replenished with the fresh medium. At 8 h post-infection (p.i.), the cells were harvested for detecting the viral NS1 protein by immunoblot analysis.

### Chemotaxis assay

As galectin-3 is a chemoattractant for monocytes and macrophages [24, 33], we employed the chemotaxis assay to validate the bioactivity of recombinant mouse galectin-3 proteins. We examined the migratory capabilities of RAW264.7 cells in response to galectin-3. The cells that had been starved for 12 h were placed in the upper compartment of the Boyden chamber (NeuroProbe, Cabin John, MD, USA) and allowed to migrate through the 8-µm-pore polycarbonate membranes (NeuroProbe) into the lower compartment filled with various concentrations of recombinant galectin-3 proteins that served as the chemoattractant. After being incubated for 6 h, the cells that migrated through the membrane to the lower surface were fixed by methanol, stained with Giemsa, and counted under a microscope. The number of migratory cells was the average of the cells counted in three randomly selected fields in each well.

### Colorimetric binding assay

An ELISA-based method was used to evaluate the interaction between galectin-3 or galectin-1 and influenza virus as described previously [11]. Influenza viruses (4 hemagglutinating unit (HAU)/well) were coated on 96-well plates as capture elements. Subsequently, various amounts of recombinant galectin-3 or galectin-1 protein were added to the wells and incubated at 4 °C overnight. Biotinylated goat anti-galectin-1 antibody (BAF1152, R&D) or mouse anti-galectin-3 antibody (sc32790, 1:1000, Santa Cruz) was added and incubated at room temperature for 2 h, followed by addition of HRP-conjugated streptavidin (DY998, 1:200, R&D) or goat anti-mouse HRP antibody (115-035-003, 1:200, Jackson Immuno Research) for an additional 20 min at room temperature. The plates were developed with 3,3′,5,5′-tetramethyl benzidine (KPL, Baithersburg, MD, USA). The enzyme reaction was stopped with 2 N H_2_SO_4_ after incubation for 20 min at room temperature, and the absorption was measured at 450 nm.

### Hemagglutination inhibition assay

Hemagglutination inhibition test was used to verify the binding of influenza virus to galectin-3 as previously described [11]. IAV (2 HAU) was incubated with various concentrations of recombinant mouse galectin-3 proteins at room temperature for 60 min, followed by addition of 0.8% human O-type erythrocyte suspension from outdated blood provided by Department of Pathology, NCKU Hospital (Tainan, Taiwan). After an additional 60-min incubation at room temperature, the presence or inhibition of hemagglutination was recorded.

### Statistical analysis

Data are expressed as mean ± standard deviation (SD). Statistical differences were compared by one-way ANOVA with Bonferroni post hoc test among three or more groups or Student’s *t* test between two groups. Survival analysis was performed using the Kaplan-Meier survival curve and log-rank test. Statistical differences were considered significant if *p* values were < 0.05. Statistical tests were performed using GraphPad Prism (version 8.0, GraphPad software, San Diego, CA, USA).

## Results

### Galectin-3 expression is upregulated in the airway of IAV-infected mice

**Fig. 1:**
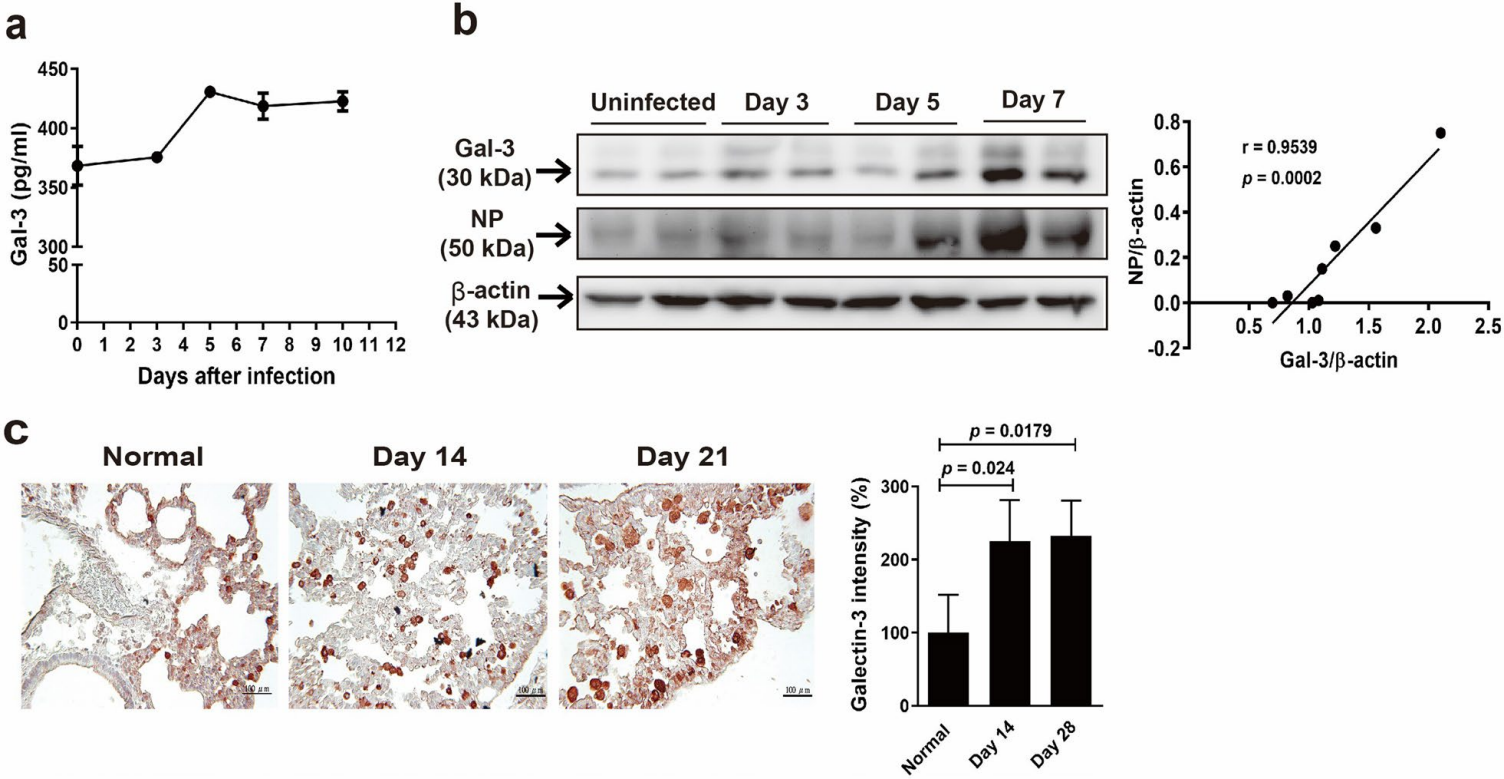
Galectin-3 is upregulated in the BAL fluid and lungs of mice following IAV infection. Mice were intranasally (**a**, **b**) and intratracheally (**c**) inoculated with IAV (10^5^ PFU/mouse) at day 0, and the BAL fluid (**a**, **b**) and lung tissue (**c**) were collected at different time points, respectively. **a** Detection of galectin-3 levels in the BAL fluid by ELISA. Values shown are mean ± SD (n = 3). **b** Detection of galectin-3 and the viral NP in the lung of individual mice by immunoblotting (left). Each lane represents samples from individual mice. Expression of β-actin served as the loading control. Pearson correlation analysis for the correlation of galectin-3 and viral NP levels showing the best-fit linear trend line (right). **c** Immunohistochemical detection (original magnification ×200; scale bar = 100 μm) (left) and quantitation of immunoreactive intensity (right) of galectin-3 with AEC (red color) as the chromogen in the lung at the recovery stage of IAV infection, with the value of normal mice arbitrarily set to 100. Values shown are mean ± SD (n = 4)

To investigate the potential association of galectin-3 with influenza virus, we determined galectin-3 levels and viral loads in the BAL fluid and lung tissue of mice infected with influenza A/WSN/33 viruses. In the BAL fluid, the amount of galectin-3 of 30 kDa was upregulated following viral infection, as examined by ELISA (Fig. 1a) and immunoblotting (Additional file 1: Fig. S1a). Quantitation of band intensities on immunoblots validated a significant increase in galectin-3 levels in the BAL fluid at day 7 p.i. (Additional file 1: Fig. S1b). These results confirm previous findings showing that levels of galectin-3 increased gradually and peaked at day 10 p.i. in the BAL fluid of mice infected with influenza A/PR/8/34 viruses [15]. As shown in Fig. 1b, immunoblot analysis of the lung tissue revealed a concomitant increase in the expression of galectin-3 and viral NP proteins, one of the major viral structural proteins expressed early after viral infection [34]. Detection of galectin-3 proteins in the mouse samples revealed that galectin-3 displayed one prominent band at 30 kDa and another faint band around 35 kDa (Additional file 1: Fig. S1a, 1b). Our results are in accordance with previous reports showing that multiple galectin-3 isoforms are produced by various cell types. We therefore presume that the two bands corresponded to isoforms of mouse galectin-3. Based on the best-fit linear trend line, we found a positive correlation (*r* = 0.9539, *p* = 0.0002) between levels of galectin-3 and viral NP proteins in the lung tissue during the acute phase of viral infection (Fig. 1b, right). Results from immunohistochemistry (Fig. 1c, left) and quantitative analysis (Fig. 1c, right) of the lung tissue also revealed higher galectin-3 immunoreactivity during the recovery phase at days 14 and 21 p.i. compared to the uninfected normal lung tissue. Collectively, these results indicate that galectin-3 expression is increased in the BAL fluid and lung tissue during both acute and recovery phases of IAV infection.

### Mice lacking galectin-3 are resistant to IAV infection

**Fig. 2:**
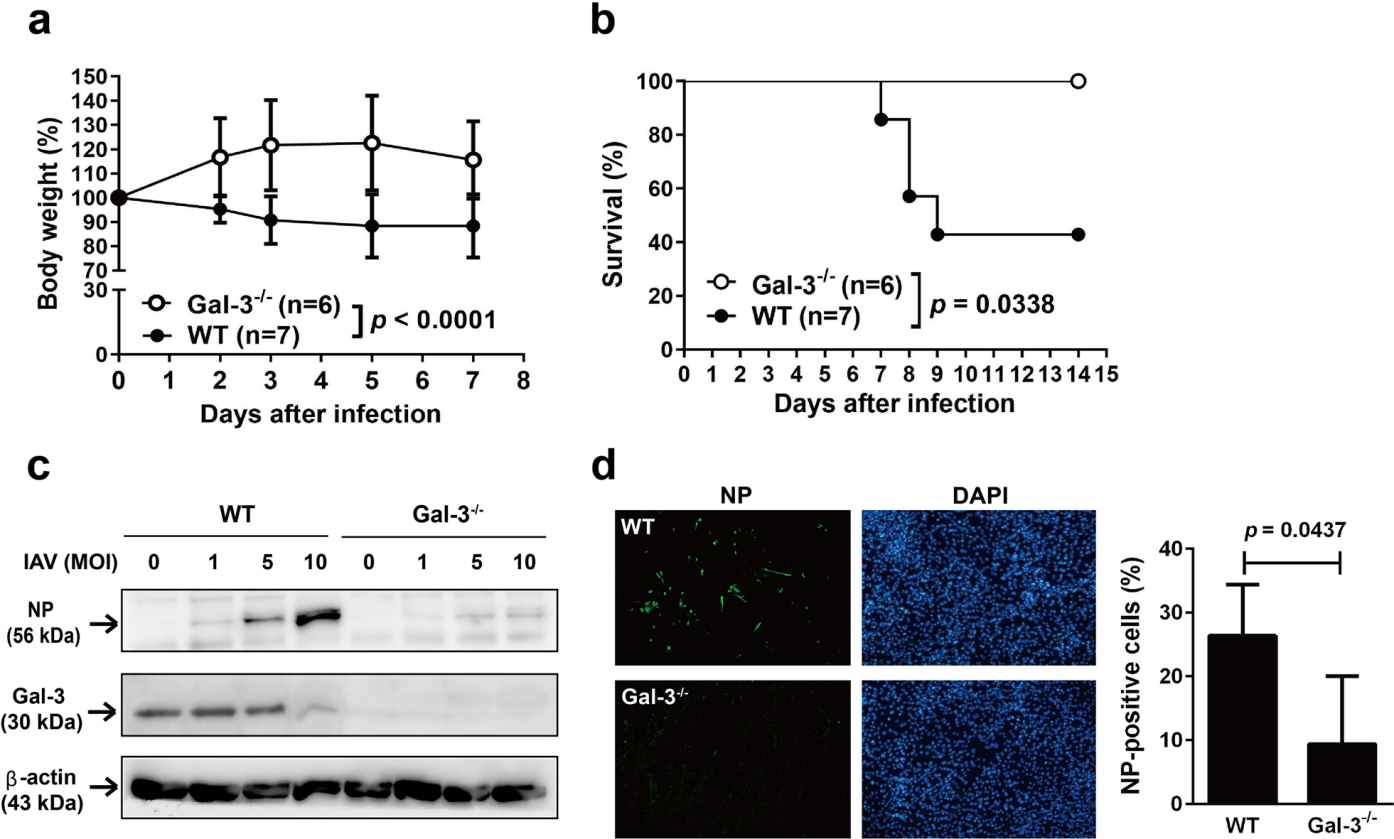
Deficiency in galectin-3 reduces mouse lethality after IAV challenge and decreases susceptibility of mouse macrophages and lung fibroblasts to IAV infection. **a**, **b** Gal-3^−/−^ and WT mice were intranasally inoculated with 10^5^ PFU of IAV at day 0. Changes in body weights (from day 0 to day 7) expressed as the percentage of pre-infection (day 0) body weight (**a**) and Kaplan-Meier survival curves (**b**). **c** Detection of the viral NP in the macrophages of Gal-3^−/−^ and WT mice infected with IAV. BMDMs were infected with IAV at MOI of 1, 5, and 10, or mock-infected for 24 h. Levels of viral NP and galectin-3 proteins in the cell lysates were examined by immunoblotting. Expression of β-actin served as the loading control. **d** Immunofluorescence detection (left) and quantitation of immunoreactive intensity (right) of the viral NP in lung fibroblasts of Gal-3^−/−^ and WT mice infected with IAV. Primary lung fibroblasts from both mice were infected with IAV at an MOI of 5 for 24 h. Nuclei were stained with DAPI. Values shown are mean ± SD (n = 4)

To study the role of galectin-3 in host defense against influenza virus infection, we compared the survival curves and body weight changes between Gal-3^−/−^ and WT mice after intranasal infection with IAV. As shown in Fig. 2a, IAV-infected WT mice continued to lose more weight over time compared with their Gal-3^−/−^ counterparts. Analysis of the entire body weight curves from day 0 through day 7 while all the infected mice were still alive revealed that WT mice significantly lost more weight than Gal-3^−/−^ mice (*p* < 0.0001). Kaplan-Meier survival curve analysis indicates that four of seven WT mice succumbed to the sublethal dose of IAV between days 7 and 9 p.i., whereas all Gal-3^−/−^ mice recovered from the infection and survived for the duration of the experiment (14 days) (*p* = 0.0338) (Fig. 2b). Thus, deficiency in galectin-3 protects mice against IAV infection.

### Cells deficient in galectin-3 reduces, whereas cells overexpressing galectin-3 enhances, IAV infection

**Fig. 3:**
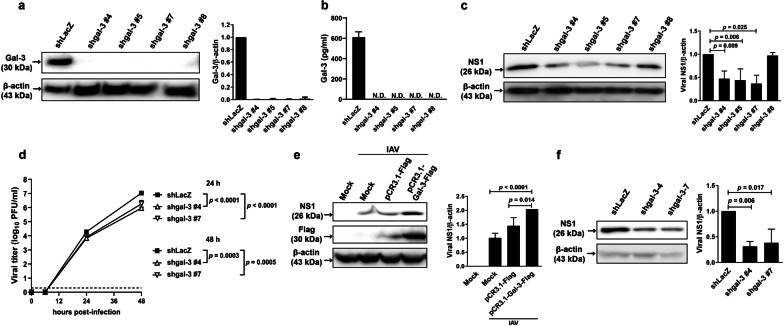
Knockdown of galectin-3 decreases viral production in IAV-infected A549 cells. **a** Examination (left) and quantitation (right) of the knockdown efficiency of different shRNAs specific to human galectin-3. **b** Quantification of galectin-3 in the conditioned medium of galectin-3-knockdown and vector control A549 cells infected with IAV by ELISA (n = 3). **c** Detection (left) and quantitation (right) of the viral NS1 protein in different galectin-3-knockdown and control cells following IAV infection. Values shown are mean ± SD (n = 3–4). **d** Quantification of viral titers in galectin-3-knockdown and control cells infected with IAV for 24 and 48 h by the plaque assay (n = 3). A549 cells were transduced with lentiviral vectors encoding different shRNAs specific to galectin-3 or LacZ to generate stable knockdown cell clones and then infected with Influenza A/WSN/33 (H1N1) viruses at MOI of 1 for 7 h (**a**, **c**) and 0.01 for 24 and 48 h (**d**). Levels of galectin-3 (**a**) and viral NS1 proteins (**c**) were examined by immunoblotting. Expression of β-actin served as the loading control. **e** Detection (left) and quantitation (right) of the viral NS1 protein in galectin-3-overexpressing and control A549 cells after infection with IAV. Cells that had been transfected with the Flag-tagged galectin-3 expression vector, the control vector, or left untransfected were infected with IAV at an MOI of 1 for 7 h and then examined for viral NS1 and galectin-3 proteins by immunoblotting. Expression of β-actin served as the loading control. **f** Detection (left) and quantitation (right) of the viral NS1 protein in galectin-3-knockdown and control A549 cells after infection with influenza A/PR/8/34 (H1N1) viruses at an MOI of 1 for 7 h. Expression of β-actin served as the loading control. Values shown are mean ± SD (n = 3). Ratios of shLacZ control cells (**a**, **c**, **f**) and IAV-infected mock cells (**e**) were arbitrarily set to 1

To explore whether resistance of Gal-3^−/−^ mice against lethal influenza infection was attributable to reduced viral production, we infected BMDMs and lung fibroblasts of Gal-3^−/−^ and WT mice with IAV and compared their viral production levels. Immunoblot analysis shows that levels of the NP protein in Gal-3^−/−^ BMDMs were much lower than those in their WT counterparts (Fig. 2c). Results of immunofluorescence microscopy (Fig. 2d, left) and quantitative analysis (Fig. 2d, right) of the lung fibroblasts revealed that percentages of NP-positive cells were lower in Gal-3^−/−^ cells than in WT cells. To further confirm the involvement of galectin-3 in the enhancement of IAV infection, we silenced galectin-3 expression in A549 cells via lentivirus-mediated delivery of shRNA specific to galectin-3, which was confirmed by immunoblot analysis with quantification of band intensities (Fig. 3a). Notably, vector control cells transduced with the control shRNA (shLacZ) secreted approximately 600 pg of galectin-3 from the conditioned medium of 3 × 10^5^ cells that had been cultured for 48 h (Fig. 3b). However, no detectable levels of galectin-3 were found in the conditioned medium collected from galectin-3-knockdown A549 cells by ELISA with the detection limit of 62.5 pg/ml (Fig. 3b). These results collectively indicate that lentivirus-mediated shRNA knockdown of galectin-3 in A549 cells abrogates not only intracellular, but also extracellular galectin-3 expression. Except for the #8 clone, galectin-3-knockdown clones significantly produced lower levels of the viral NS1 protein, which is preferentially synthesized at early times of infection [34], at 7 h p.i. (Fig. 3c) and generated lower viral titers at 24 and 48 h (Fig. 3d) compared to vector control cells. To exclude the potential off-target effects of lentiviral transduction, we overexpressed galectin-3 in A549 cells by plasmid transfection. As shown in Fig. 3e, cells transiently transfected with pCR3.1-Gal-3-Flag produced higher levels of the NS1 protein than those transfected with the control plasmid pCR3.1-Flag. Moreover, levels of the NS1 protein produced from virus-infected cells were not significantly different between cells transfected with pCR3.1-Flag and untransfected cells. We also used another H1N1 strain to confirm the enhancing effect of galectin-3 on IAV infection. A549 cells transduced with shRNA specific to galectin-3 expressed lower levels of the NS1 protein compared with those transduced with the control vector shLacZ following infection with influenza A/PR/8/34 viruses at 7 h p.i. (Fig. 3f). Thus, galectin-3-mediated enhancement of influenza virus infection is not restricted to influenza A/WSN/33 viruses. Taken together, these results indicate that intracellular galectin-3 enhances IAV infection.

### Intracellular galectin-3 has no effects on viral binding and internalization into lung epithelial cells

**Fig. 4:**
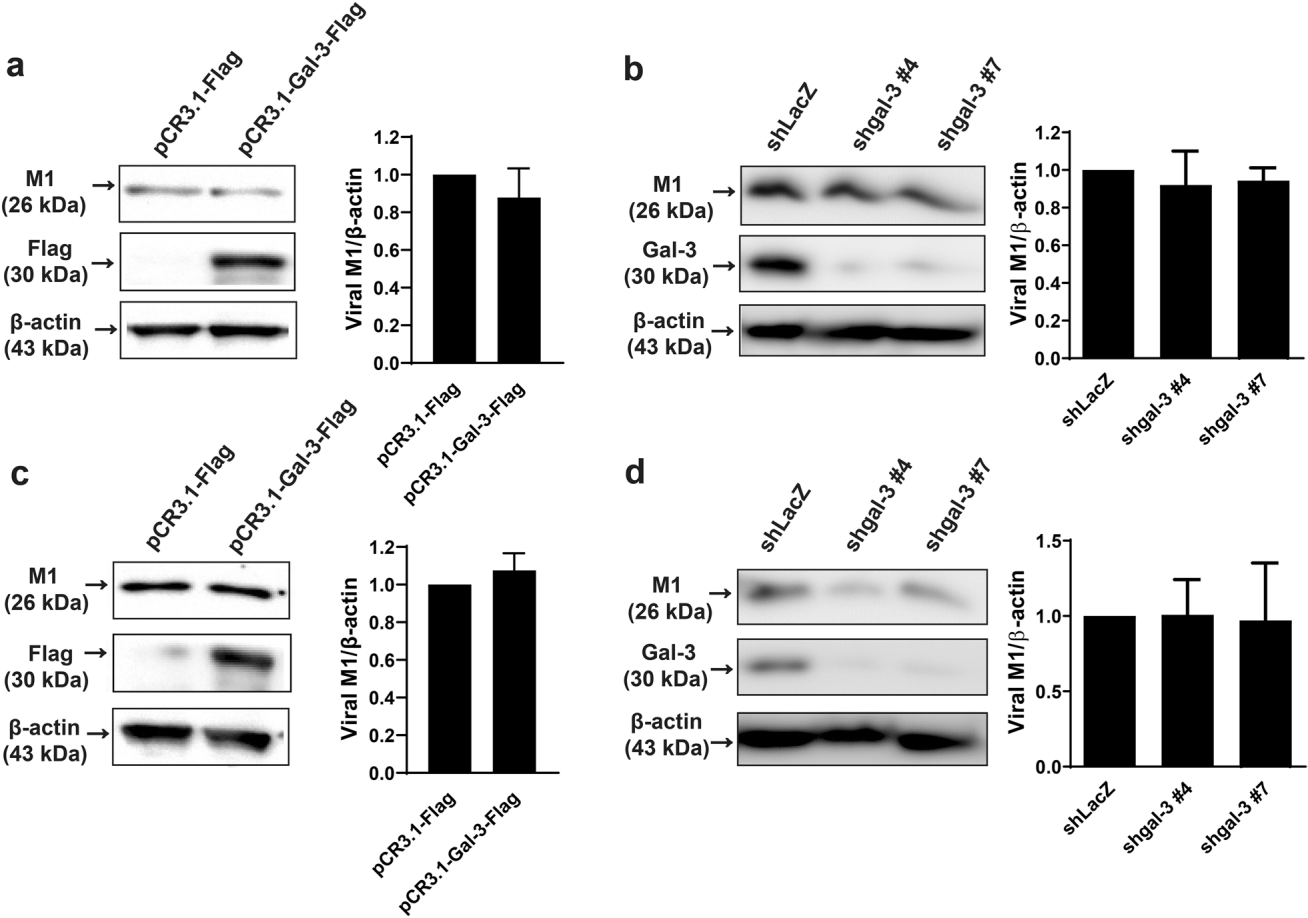
Changes in intracellular galectin-3 levels fail to affect viral binding and internalization into A549 cells. **a–d** Galectin-3-overexpressing (**a**, **c**) or knockdown (**b**, **d**) A549 cells and their vector control cells were infected with IAV at an MOI of 5 at 4 °C for 60 min and washed by ice-cold PBS for the viral attachment assay (**a**, **b**) or at 37 °C for 30 min and washed by acid PBS (pH 1.3) for the internalization assay (**c**, **d**). Detection (left) and quantitation (right) of the viral M1 and galectin-3 were examined by immunoblotting. Expression of β-actin served as the loading control. Values shown are mean ± SD (n = 3–4). Ratios of vector control cells (**a–d**) were arbitrarily set to 1

Given that knockdown of galectin-3 reduced viral protein levels and viral titers (Fig. 3), we next used overexpression and knockdown approaches to determine which stage(s) of the viral life cycle was affected by galectin-3. Galectin-3-overexpressing or knockdown A549 cells and their control counterparts were incubated with IAV at 4 °C for 60 min (for the binding assay) and 37 °C for 40 min (for the internalization assay) and subsequently washed with ice-cold PBS and acid PBS to eliminate the unbound and uninternalized viral particles, respectively. Detection of viral M1 protein, which is the most abundant viral protein in the virion and commonly used as a marker for initial viral entry [35], revealed that the amounts of bound (Fig. 4a and b) or internalized (Fig. 4c and d) viral particles in galectin-3-overexpressing or knockdown cells were similar to those in the control cells [35, 36]. Collectively, these results demonstrate that intracellular galectin-3 does not affect the binding and internalization during viral entry into lung epithelial cells.

### Intracellular galectin-3 promotes nuclear import of the vRNP complex

**Fig. 5:**
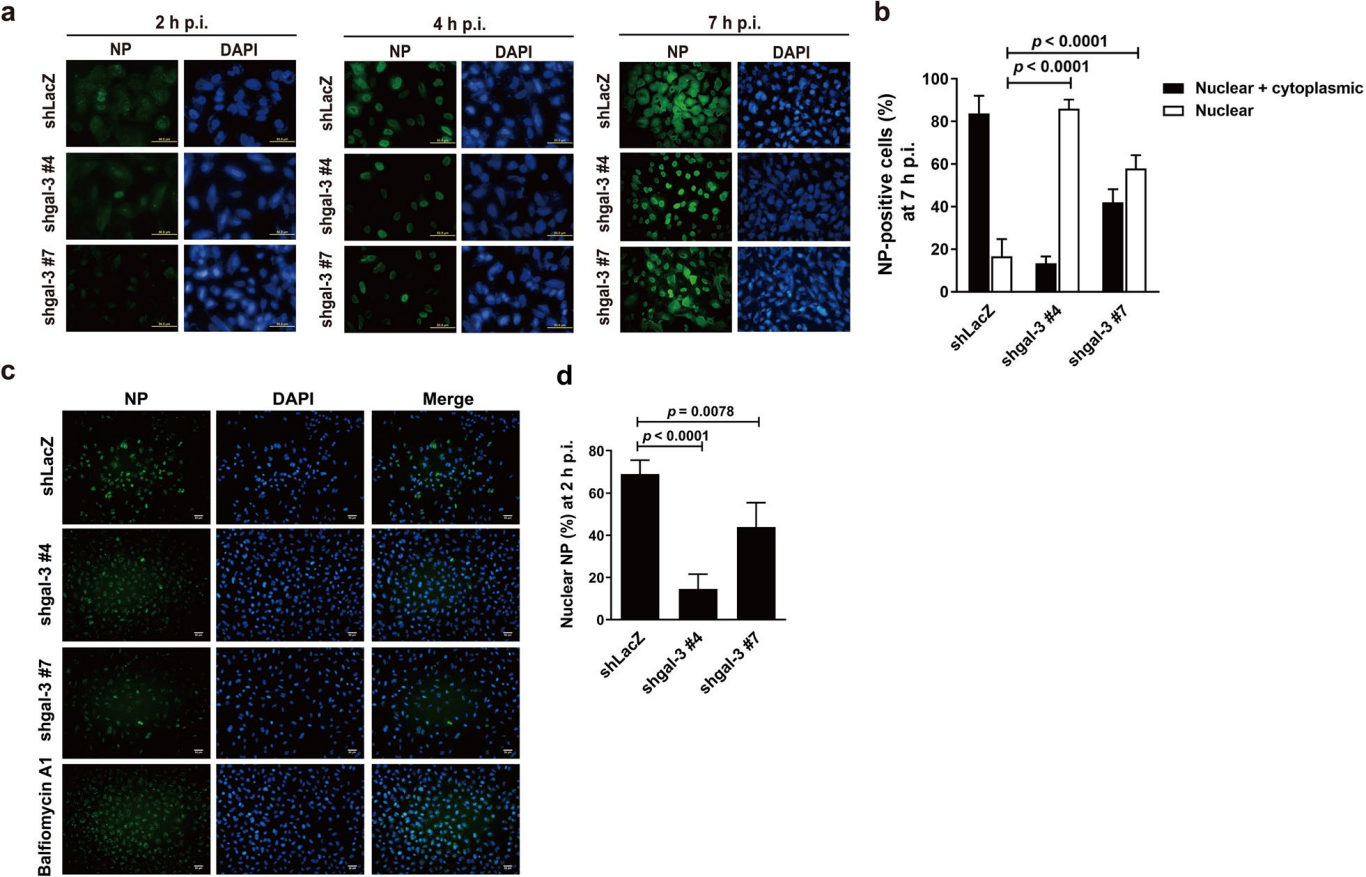
Knockdown of galectin-3 reduces the nuclear import of the vRNP complex. **a**, **b** Galectin-3-knockdown and vector control A549 cells were infected with IAV at an MOI of 5 for 60 min, fixed, and then permeabilized at 2 h, 4 h, and 7 h p.i. for immunofluorescence staining with the anti-NP antibody. Nuclei were stained with DAPI. Localization of the viral NP indicative of the vRNP complex (**a**) and quantitative analysis of nucleocytoplasmic distribution of the NP at 7 h p.i (n = 3–5) (**b**). Representative images are shown (original magnification ×400, scale bar = 50 μm). **c**, **d** Galectin-3-knockdown and vector control A549 cells were infected with IAV at an MOI of 100 for 60 min in the presence of cycloheximide (100 µg/ml), fixed, and then permeabilized at 2 h p.i. for immunofluorescence staining with the anti-NP antibody. Nuclei were stained with DAPI. IAV-infected A549 cells in the presence of bafilomycin A1 (100 nM) were used as a positive control for inhibition of vRNP nuclear import. Localization of the viral NP (**c**) and quantitative analysis of nuclear localization of the NP at 2 h (n = 4) (**d**). Representative images are shown (original magnification ×200, scale bar = 50 μm). Due to different infectivity of A549 cells transduced with different shRNAs, NP-positive (green staining) cells were examined for their nuclear/cytoplasmic localization at 7 h p.i. (**a**, **b**) or nuclear localization at 2 h p.i. (**c**, **d**). The NP observed in both the nucleus and cytoplasm is denoted as Nuclear + cytoplasmic, whereas the NP observed only in the nucleus is denoted as Nuclear

Next, we studied the fate of the internalized virus by examining the cellular distribution of IAV after viral entry. As replication of IAV takes place in the nucleus of infected cells, efficient dynamic intranuclear traffic and export of the vRNP towards the cytoplasm to allow production of infectious virions are required [37]. Given that the viral NP, which is associated with viral genomic RNA, can be regarded as a marker of vRNP localization, we examined the subcellular localization of vRNP in galectin-3-knockdown and vector control A549 cells infected with IAV at 2, 4, and 7 h p.i. by immunofluorescence staining for the viral NP. Typically, vRNP is confined to the nucleus at the early stage of infection and enters the cytoplasm for packaging into progeny virions at the late stage of infection. We found that the viral NP was hardly detectable at 2 h p.i. in all groups of cells (Fig. 5a). In control cells, it was localized in the nucleus at 4 h p.i. and was observed abundantly in the cytoplasm at 7 h p.i. (Fig. 5a). At 4 h p.i. in galectin-3-knockdown cells, the viral NP was detectable in the nucleus, to a similar degree as the vector control cells. However, at 7 h p.i., 60–80% of galectin-3-knockdown, NP-positive cells contained the nuclear NP, whereas only about 15% of NP-positive, vector control cells showed positive nuclear staining for the NP (Fig. 5a, b). While 84% of NP-positive control cells expressed the NP in both the nucleus and cytoplasm, only 13–42% of galectin-3-knockdown cells contained both nuclear and cytoplasmic NP. Taken together, these results suggest that galectin-3 may enhance nuclear import and/or export of the vRNP complex.

Next, we investigated whether galectin-3 affected nuclear import of the vRNP. Galectin-3-knockdown and vector control A549 cells were infected with IAV at an MOI of 100 for 60 min in the presence of cycloheximide, a widely used eukaryotic protein synthesis inhibitor, to inhibit de novo synthesis of the viral NP. We also used bafilomycin A1 as a positive control for inhibiting vRNP nuclear import. Localization of the vRNP was detected by immunofluorescence staining for the viral NP at 2 h p.i. (Fig. 5c). In vector control cells, 70% of infected cells showed clearly nuclear localization, whereas only 15–40% of galectin-3-knockdown cells expressed the NP in the nucleus (Fig. 5d). Taken together, these results indicate that intracellular galectin-3 promotes nuclear import of the vRNP. However, we cannot exclude the possibility that galectin-3 also impacts vRNP nuclear export at the late stage of the viral life cycle.

### Intracellular galectin-3 enhances the RdRp activity and hence increases viral RNA synthesis

**Fig. 6:**
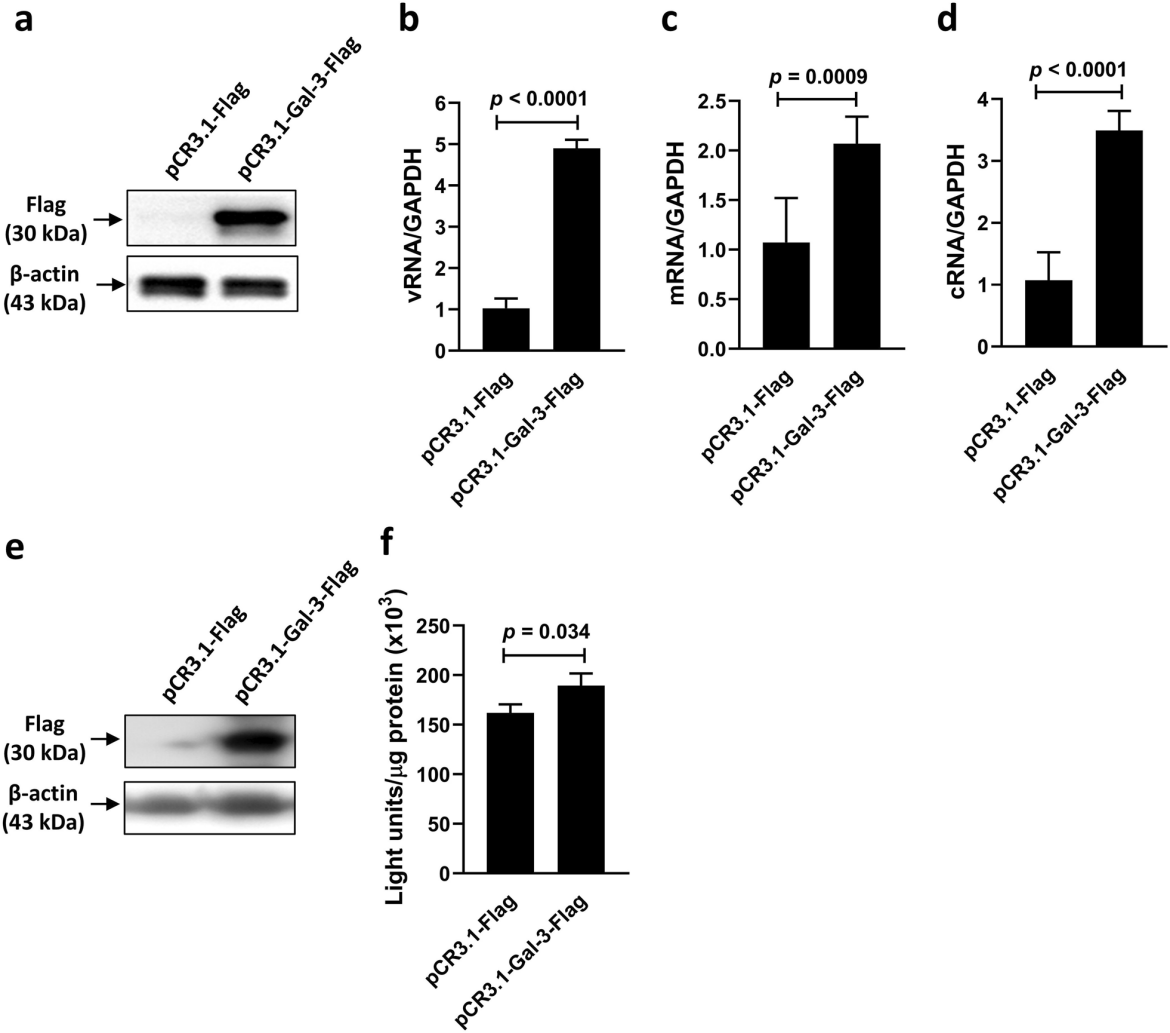
Overexpression of galectin-3 enhances the RdRp activity of IAV. **a–d** A549 cells that had been transfected with the Flag-tagged galectin-3 expression vector or the control vector were infected with IAV at an MOI of 1 for 5 h. Detection of Flag-tagged galectin-3 proteins by immunoblotting with the anti-Flag antibody in A549 cells (**a**). Expression of β-actin served as the loading control. Quantification of vRNA (**b**), mRNA (**c**), and cRNA (**d**) of the viral NP by RT-qPCR and normalization to GAPDH mRNA levels (n = 3). Ratios of vector control cells (**b–d**) were arbitrarily set to 1. **e**,** f** Galectin-3-overexpressing and vector control 293T cells were cotransfected with pPolI-Luc and four expression vectors individually encoding IAV proteins (PA, PB1, PB2, and NP) for assessing viral polymerase activity by the minireplicon assay. Luciferase activities were determined at 48 h post-transfection and expressed as light units/µg protein (n = 3). Detection of Flag-tagged galectin-3 proteins by immunoblotting with the anti-Flag antibody (**e**) and viral polymerase activity (**f**) in 293T cells

To further investigate the effects of galectin-3 overexpression or knockdown on viral RNA synthesis in A549 cells, we used strand-specific RT-qPCR to quantify the three viral RNA classes (vRNA, mRNA, and cRNA) of the viral NP in IAV-infected cells. Immunoblot analysis confirmed galectin-3 overexpression in A549 cells transfected with the eukaryotic expression vector pCR3.1-Gal-3-Flag (Fig. 6a). Upregulation of galectin-3 in A549 cells resulted in a 2- to 4-fold increase in the synthesis of vRNA (Fig. 6b), mRNA (Fig. 6c), and cRNA (Fig. 6d) of the viral NP. Next, we used the minireplicon assay to measure the impact of galectin-3 overexpression on viral transcription and replication in the highly transfectable 293T cells [5]. Elevated galectin-3 expression in 293T cells transfected with pCR3.1-Gal-3-Flag was validated with immunoblot analysis (Fig. 6e). Galectin-3-overexpressing and control 293T cells were cotransfected with pPolI-Luc and four expression vectors individually encoding the influenza viral proteins PA, PB1, PB2, and NP for 48 h. The reporter plasmid pPolI-Luc encodes firefly luciferase that was flanked by conserved 5′ and 3′ untranslated regions of influenza RNA genome segments and under the control of the RNA polymerase I promoter. Thus, the levels of firefly luciferase represent the transcription and replication activities of the viral polymerase complex. Figure 6f shows that luciferase activities were significantly higher in galectin-3-overexpressing cells than in control cells transfected with pCR3.1-Flag.

**Fig. 7:**
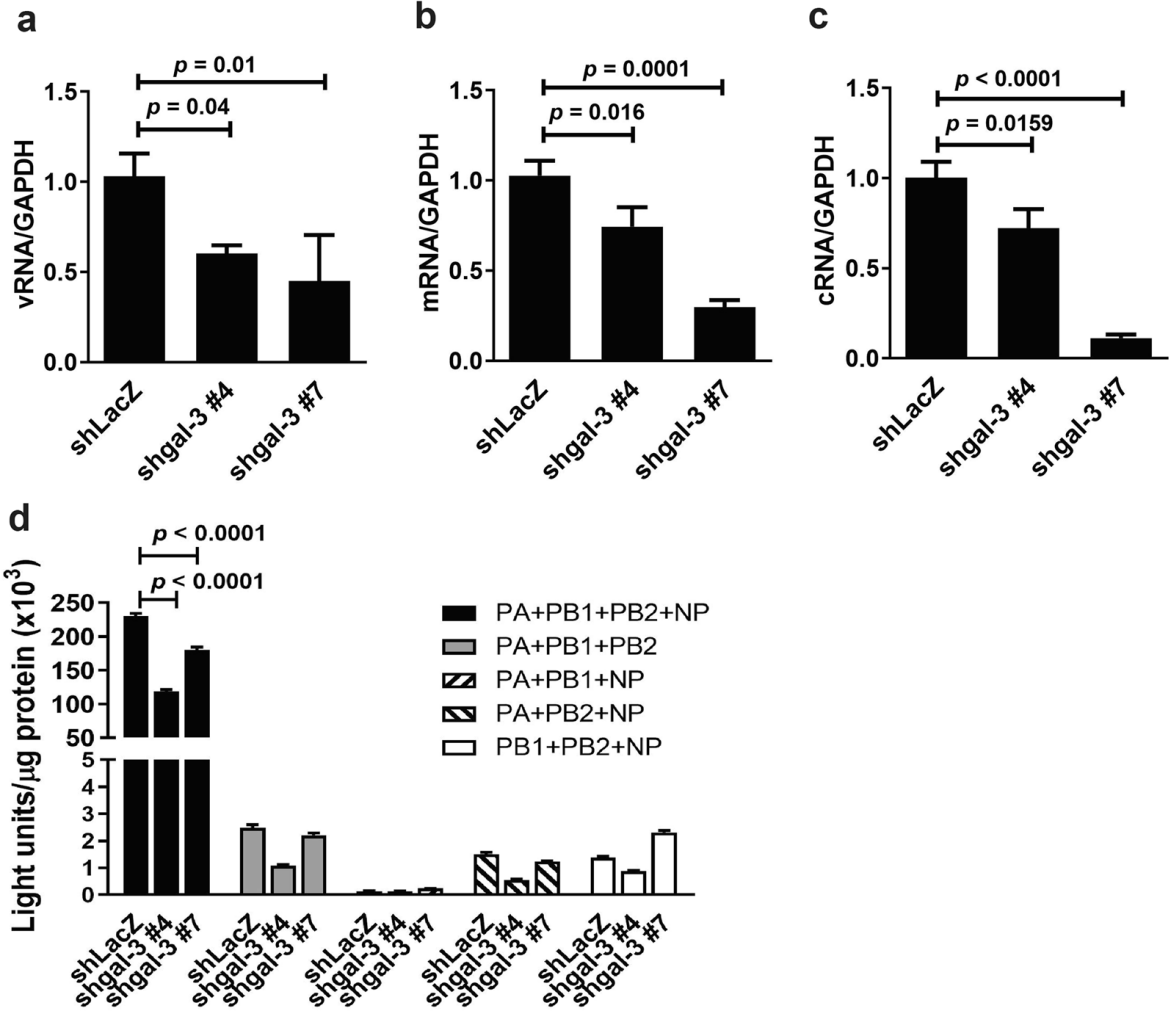
Knockdown of galectin-3 decreases the RdRp activity of IAV. **a–c** Galectin-3-knockdown and vector control A549 cells were infected with IAV at an MOI of 1 for 7 h. Quantification of vRNA (**a**), mRNA (**b**), and cRNA (**c**) of the viral NP by RT-qPCR and normalization to GAPDH mRNA levels (n = 3). Ratios of shLacZ vector control cells were arbitrarily set to 1. **d** Detection of viral polymerase activity by the minireplicon assay. Galectin-3-knockdown and vector control 293T cells were cotransfected with pPolI-Luc and four expression vectors individually encoding IAV proteins (PA, PB1, PB2, and NP). At 48 h post-transfection, their luciferase activities were determined and expressed as light units/µg protein (n = 3)

Having shown that overexpression of galectin-3 increased the RdRp activity and enhanced viral RNA synthesis, we further used galectin-3 knockdown cells to validate these effects. Galectin-3-knockdown A549 cells, confirmed by immunoblot analysis (Fig. 4b), generated lower amounts of vRNA (Fig. 7a), mRNA (Fig. 7b), and cRNA (Fig. 7c) of the viral NP compared with the control cells. We also examined whether knockdown of galectin-3 decreased viral transcription and replication with the minireplicon assay. As shown in Fig. 7d, higher luciferase activities were detected in control shLacZ cells cotransfected with the four IAV expression vectors (PA, PB1, PB2, and NP) and pPolI-Luc, whereas only very low or negligible activities were found in those cotransfected with any three IAV expression vectors and pPolI-Luc. These data confirm that the three components of the RdRp and the NP are required for the RdRp activity. Notably, luciferase activities were decreased 2-fold in galectin-3-knockdown cells compared with those in control shLacZ cells after cotransfection of the four IAV expression vectors and pPolI-Luc (Fig. 7d). The results from overexpression and knockdown experiments collectively indicate that galectin-3 enhances the RdRp activity required for viral mRNA transcription and genome replication.

### Intracellular galectin-3 interacts with the PA subunit of the RdRp

**Fig. 8:**
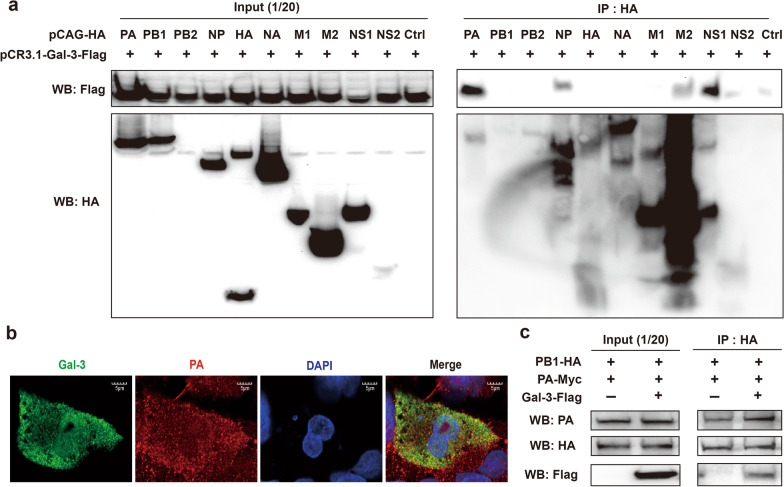
Galectin-3 interacts with the PA subunit of the RdRp. **a** The association of galectin-3 with viral proteins. 293T cells were cotransfected with expression vectors encoding Flag-tagged galectin-3 and the indicated HA-tagged IAV proteins. The cell lysates were prepared for immunoprecipitation with anti-HA agarose. The cell lysates (input) and the immunoprecipitates were subjected to immunoblot analysis with anti-HA or anti-Flag antibody. **b** Colocalization of galectin-3 with the PA subunit in IAV-infected cells. A549 cells were transfected with pCR3.1-Gal-3-Flag for 48 h and then infected with IAV at an MOI of 5 for 8 h. Cells were then fixed and permeabilized for immunofluorescence staining with anti-PA (red) and anti-Flag (green) antibodies. Nuclei were stained with DAPI. Representative images are shown (original magnification ×900, scale bar = 5 μm). **c** Association of PA subunit with PB1 subunit in the presence of galectin-3. 293T cells were cotransfected with expression vectors encoding the Flag-tagged galectin-3, HA-tagged PB1, and Myc-tagged PA. Whole cell lysates without immunoprecipitation (5% input) were estimated for the expression levels of PA, PB1, and galectin-3 (left). Cell lysates were used for immunoprecipitation with the anti-HA agarose. Cell lysates (input) and immunoprecipitates were subjected to immunoblot analysis with anti-HA, anti-PA, and anti-Flag antibodies. IP: immunoprecipitation; WB: western blotting

To investigate whether galectin-3-mediated enhancement of viral transcription and replication was attributable to its direct interaction with viral proteins, we performed the co-immunoprecipitation assay in 293T cells. Cells were cotransfected with pCR3.1-Gal-3-Flag encoding Flag-tagged galectin-3 and one of the pCAG-HA expression vectors encoding HA-tagged viral proteins (PA, PB1, PB2, HA, NA, NP, M1, M2, NS1, and NS2) or the HA-tagged control vector. As shown in Fig. 8a, the viral PA and NS1 proteins predominantly coprecipitated with galectin-3 compared to the remaining viral proteins. We also used IAV-infected cells to examine whether galectin-3 was associated with the PA subunit. A549 cells were transfected with the galectin-3 expression vector pCR3.1-Gal-3-Flag, followed by infection with IAV. Confocal microscopy demonstrates colocalization of galectin-3 with the viral PA (Fig. 8b). The N-terminal domain of the PA subunit contains the endonuclease activity required for cap-snatching, whereas its C-terminal domain interacts with the N-terminal domain of the PB1 subunit, leading to RNA polymerase activity. In addition to cap-snatching, the PA is essential for the PB1 binding to the 5′ end of the vRNA promoter and required for the nuclear accumulation of the PB1 [38, 39]. Direct interaction between galectin-3 and PA/PB1 proteins was further confirmed by the co-immunoprecipitation assay, followed by immunoblotting of 293T cells that had been cotransfected with three plasmids individually expressing Flag-tagged galectin-3, HA-tagged PB1, and Myc-tagged PA. As shown in Fig. 8c, overexpression of galectin-3 increased the interaction of PA and PB1 subunits (top lane, right panel). Furthermore, immunoprecipitation with the anti-HA antibody and immunoblotting with the anti-Flag antibody of the cotransfected cells revealed that the amount of the PB1 pulled down along with galectin-3 in galectin-3-overexpressing cells was increased compared to the cells without galectin-3 overexpression (bottom lane, right panel). However, the amount of the PB1 was similar regardless of galectin-3 overexpression (middle lane, right panel), which excluded the possibility of higher PB1 amounts in galectin-3-overexpressing cells. Taken together, these results indicate that galectin-3 directly interacts with the PA subunit, but not the PB1 subunit. Nevertheless, galectin-3 can indirectly associate with the PB1 subunit through the PA-PB1 interaction. Strong and mild interactions between galectin-3 and the viral NS1 as well as between galectin-3 and the viral NP were also observed, respectively (Fig. 8a). Our results demonstrate that galectin-3 can facilitate nuclear import of the vRNP complex and viral RNA synthesis (Figs. 5, 6 and 7), suggesting that the viral NP or NS1 protein may participate in these processes through binding to galectin-3. In conclusion, our results indicate that galectin-3 promotes viral RNA synthesis through association with the PA subunit and enhancement of the formation of the PA-PB1 dimeric complex.

### Extracellular galectin-3 enhances IAV infection

Galectin-3 possesses diverse intracellular and extracellular functions. Given that galectin-3 was upregulated intracellularly and extracellularly in the airway during IAV infection (Fig. 1), we further tested the impact of exogenously added galectin-3 on IAV infection in A549 cells. We generated recombinant mouse galectin-3 proteins and validated their biological activity of enhancing macrophage migration [33]. The Boyden chamber assay revealed that purified mouse recombinant galectin-3 proteins enhanced the migration of mouse RAW264.7 macrophages (Additional file 1: Fig. S2a, S2b). Next, we examined whether galectin-3 had similar biological activities as galectin-1 [11]. While galectin-1 bound to IAV coated on a solid phase in a dose-dependent manner at concentrations ranging from 6.25 to 100 µg/ml, galectin-3 bound to influenza virus to a much lesser extent than galectin-1 (Additional file 1: Fig. S2c). Furthermore, galectin-3 at concentrations ≥ 62.5 µg/ml could inhibit the hemagglutination activity of IAV (Additional file 1: Fig. S2d), which was 40-fold weaker than galectin-1. Notably, galectin-3 per se with concentrations as high as 100 µg/ml had no hemagglutination activity (Additional file 1: Fig. S2d). Next, we evaluated whether treatment with recombinant galectin-3 proteins could protect mice against lethal IAV infection. Contrary to the results obtained from recombinant galectin-1 proteins [11], intranasal treatment with 50 µg of recombinant galectin-3 at days 2, 3, and 5 p.i. had no effects on improving body weight (Additional file 1: Fig. S3a) nor prolonging survival time (Additional file 1: Fig. S3b) of IAV-infected mice. Collectively, these results demonstrate that unlike galectin-1, extracellular galectin-3 has no anti-influenza virus activity.

**Fig. 9:**
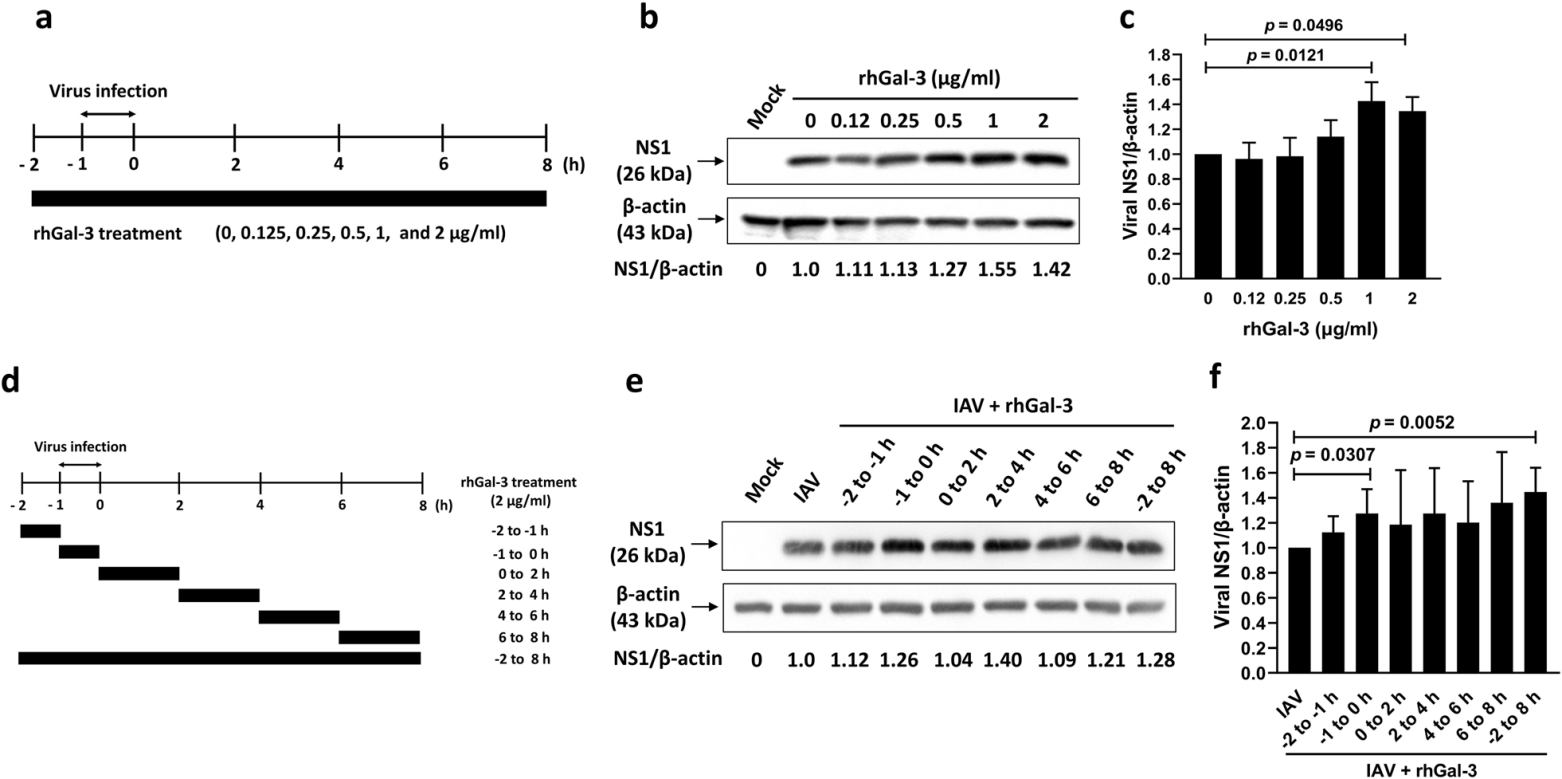
Extracellular galectin-3 enhances influenza virus infection. **a–c** A549 cells (4.2 × 10^5^) cultured in 6-well plates overnight were infected with IAV at an MOI of 1 at 37 °C for 1 h (at − 1 to 0 h). The cells were then washed with PBS for three times to remove the residual viruses and replenished with fresh serum-free DMEM containing 1 µg/ml of TPCK-trypsin at 37 °C. Human recombinant galectin-3 proteins (0.12, 0.25, 0.5, 1, and 2 µg/ml) were present throughout the whole 10-h duration of the experiment (1 h before infection, during 1 h-infection, and after infection for 8 h). Treatment schedule (**a**) as well as detection (**b**) and quantification (**c**) of the viral NS1 protein by immunoblotting at the end of the experiment. **d–f** A549 cells were infected with IAV as described in **a–c** and treated with 2 µg/ml of human recombinant galectin-3 proteins at − 2 to − 1, − 1 to 0, 0 to 2, 2 to 4, 4 to 6, 6 to 8 h, or during the whole duration (− 2 to 8 h). Treatment schedule of galectin-3 in the time-of-addition experiment (**d**) as well as detection (**e**) and quantification (**f**) of the viral NS1 protein by immunoblotting at the end of the experiment. Expression of β-actin served as the loading control. Values shown are mean ± SD (n = 3). Ratios of cells without galectin-3 treatment were arbitrarily set to 1

We further examined whether exogenously added recombinant galectin-3 proteins enhanced influenza viral infection in cell culture. A549 cells were infected with IAV for 1 h (at − 1 to 0 h) in the presence of recombinant galectin-3 proteins throughout the whole 10-h duration of the experiment (− 2 to 8 h) (Fig. 9a). Expression of the NS1 protein in IAV-infected cells treated with 1 or 2 µg/ml of galectin-3 was significantly higher than that without galectin-3 treatment, suggesting that exogenously added galectin-3 enhances influenza virus infection (Fig. 9b and c). One life cycle of influenza virus is divided into virus entry, uncoating, entry of the vRNP into the nucleus, viral genome replication and translation, and progeny virion release [4]. We performed the time-of-addition assay to determine which viral step(s) of the life cycle was affected by extracellular galectin-3 in A549 cells. Recombinant galectin-3 proteins (2 µg/ml) were applied before, during, and after IAV infection at seven different durations (− 2 to − 1, − 1 to 0, 0 to 2, 2 to 4, 4 to 6, 6 to 8, and − 2 to 8 h) (Fig. 9d), and the NS1 expression was assessed by immunoblotting. The NS1 protein was significantly increased in IAV-infected cells treated with galectin-3 during viral adsorption (− 1 to − 0 h) and throughout the whole duration (− 2 to 8 h) (Fig. 9e and f). However, there were no significant changes in NS1 levels when galectin-3 was added before viral adsorption (− 2 to − 1 h), during entry stage (0 to 2 h), during viral replication and translation stages (2 to 4 and 4 to 6 h), or at viral assembly stage (6 to 8 h) of the viral life cycle compared to those in the infected cells without galectin-3 treatment (Fig. 9e and f). Furthermore, we compared the nucleocytoplasmic distribution of the viral NP between galectin-3-treated and untreated A549 cells infected with IAV at 2, 3.5, 4.5, and 6 h p.i. by immunofluorescence staining for the NP. While the viral NP was hardly detectable at 2 h p.i., it was observed in the nucleus at 3.5 h p.i. and more abundantly at 4.5 h p.i. (Additional file 1: Fig. S4a) in both cells. The percentages of NP-positive cells expressing nuclear NP were similar regardless of galectin-3 treatment at each observation time (Additional file 1: Fig. S4a, 4b). Of note, treatment with galectin-3 significantly increased the percentage of NP-positive cells displaying both nuclear and cytoplasmic localization in A549 cells at 6 h p.i. (Additional file 1: Fig. S4c). Taken together, these data indicate that extracellular galectin-3 enhances influenza virus infection at the viral adsorption step. Furthermore, our results also suggest that galectin-3 may enhance nuclear import and/or export of the vRNP complex, thereby promoting viral infection.

## Discussion

Galectins have been implicated as amplifiers, silencers, or tuners of inflammatory responses. Galectins, such as galectin-1 and galectin-3, are upregulated after pathogen invasion and serve as a positive or negative regulator in innate defense during inflammation [40, 41]. We have reported that galectin-1 is upregulated during influenza infection, and recombinant galectin-1 proteins can bind to the virus and ameliorate IAV pathogenesis [11]. Opposing functions have been assigned for galectin-1 and galectin-3 under physiological and pathological conditions, suggesting that the balance between these two proteins may be crucial for the homeostasis of inflammatory responses [42]. In murine encephalitis models induced by Junín virus and by encephalomyocarditis virus, galectin-3 is upregulated in the activated microglia and serves as a diagnostic marker for neuronal degeneration [43, 44]. In the present study, we show that expression of galectin-3 was elevated in the airway of mice following IAV infection. Viral burden was positively correlated with galectin-3 levels in mice. Galectin-3 knockout mice were resistant to IAV infection. These results indicate that upregulation of galectin-3 during IAV infection aggravates influenza, which may be attributable to increased viral load. Galectin-3 can enhance H5N1 virus-induced lung inflammation through the promotion of NLRP3 inflammasome activation, thereby contributing to viral pathogenesis [17]. Therefore, galectin-3 can enhance influenza virus infection through enhancing viral RNA synthesis and lung inflammation.

Nuclear galectin-3 is required in pre-mRNA splicing [45]. Galectin-3 is incorporated into spliceosomes via its association with the U1 small nuclear ribonucleoprotein (snRNP) complex. It also associates with multiple snRNPs in larger complexes outside of the spliceosome [46]. Nevertheless, the role of galectin-3 in other steps of mRNA metabolism remains poorly understood. Galectin-3 can interact with heterogeneous nuclear ribonucleoprotein (hnRNP) A2/B1 in the nucleus, and the presence of galectin-3 modulates mRNA splicing and export [47]. Galectin-3 can also bind to hnRNP L to stabilize the mucin *MUC4* mRNA in the cytoplasm of cancer cells [48]. The importance of hnRNPs, which regulate splicing and nuclear export of mRNA, in IAV infection has been elucidated. The hnRNPs A1, M, L, and K can directly interact with influenza virus M1 RNA. Among these hnRNPs, hnRNP K can interact with the NS1 binding protein, a host protein previously shown to interact with the NS1 protein, leading to promoting alternative splicing of the viral M1 mRNA segment and thereby generating the M2 mRNA [49]. Moreover, hnRNP A2/B1 can directly interact with the viral NP, and its knockdown reduces viral RNA synthesis, suggesting that hnRNP A2/B1 may be regarded as a positive regulator of the activity of the vRNP [50]. However, hnRNP A2/B1 can also interact with both the viral NS1 protein and mRNA and inhibit virus replication by suppressing the nuclear export of the NS1 mRNA, suggesting that hnRNP A2/B1 may play a negative role in regulating viral replication [51]. In the present study, we show that galectin-3 promoted viral transcription and replication through interacting with the PA subunit of the RdRp. Given that galectin-3 can interact with hnRNP A2/B1 [47] and that hnRNP A2/B1 may play different roles in regulating IAV, whether hnRNPs, in particular hnRNP A2/B1 or hnRNP L, participate in galectin-3-mediated upregulation of the transcription and replication of IAV warrants further investigation. In addition, we show that interaction between galectin-3 and the viral NS1 was stronger than that between galectin-3 and the viral NP. The NP is required for vRNP nuclear import and viral transcription and replication of influenza virus [52–56]. Moreover, the NS1 protein can suppress host antiviral responses [57]. Previous studies have shown that the NS1 protein can interact with viral polymerase complex [58], promote viral M1 mRNA export [59], and upregulate viral mRNA translation [60, 61]. These findings collectively indicate that galectin-3 impacts influenza virus infection through different strategies. The mechanism underlying the association of galectin-3 with the viral NP and/or NS1 protein requires further studies.

Galectin-3 is located and capable of functioning both extracellularly and intracellularly in the cytosol and nucleus [20, 62]. They may exert differential effects depending on whether they act intracellularly or extracellularly [20]. In the current study, our results obtained from galectin-3 knockout mice as well as galectin-3-overexpressing and knockdown A549 cells demonstrate that intracellular galectin-3 can promote influenza virus infection by enhancing vRNP nuclear import, RdRp activity, and viral transcription and replication. Furthermore, we observed elevation of galectin-3 protein levels in the airway of IAV-infected mice and in the culture supernatant of IAV-infected A549 cells. These results suggest that extracellular galectin-3 may also impact influenza virus infection. Therefore, we evaluated the effects of exogenously added recombinant galectin-3 proteins on different stages of viral life cycle in A549 cells. In addition to intracellular galectin-3, extracellular galectin-3 can also promote IAV infection at the adsorption stage of the viral life cycle (Fig. 9). Moreover, extracellular galectin-3 may enhance vRNP nuclear export/export (Additional file 1: Fig. S4) probably via its interaction with cell surface proteins, such as receptor tyrosine kinases (RTKs). However, detailed mechanisms remain unclear and are worthy of further investigation. Accumulating evidence has demonstrated that many viruses exploit RTKs for their replication cycle [63]. With regard to influenza virus, RTKs and their downstream signaling pathways have been shown to be required for viral RNA synthesis, vRNP nuclear export, and virus release as well as host immune surveillance [64–67]. Among these RTKs, the role of epidermal growth factor receptor (EGFR) has been well studied in influenza virus infection. Activation of EGFR by influenza virus infection facilitates viral internalization [36]. EGFR activation also suppresses interferon regulatory factor 1-induced interferon-λ production and increases influenza virus infection in the airway epithelium [68]. Furthermore, the EGFR/ERK pathway is activated by influenza virus infection at the early stage to attenuate the antiviral innate immunity through activation of the protein tyrosine phosphatase SHP2, thus promoting viral replication [69]. A recent report has shown that FDA-approved small-molecule inhibitors of RTKs inhibit various steps of the life cycle of influenza virus in vitro and ex vivo [70]. Of note, the most potent inhibitor targeting to EGFR family kinases (EGFR, HER2, and ErbB4) impairs influenza viral entry, polymerase activity, and viral export [70]. In addition, mixed inhibitors for the platelet-derived growth factor receptor (PDGFR) and vascular endothelial growth factor receptor (VEGFR) significantly inhibit various stages of the life cycle of influenza virus [64, 70]. Taken these results into account, the signaling pathways mediated by members of the EGFR [36, 68, 69], PDGFR [71], and VEGFR [72] may be required for influenza virus infection.

The interaction between galectin-3 and RTKs is involved in numerous cellular processes [73]. Extracellular galectin-3 interacts with the EGFR and increases EGFR phosphorylation and activation, resulting in colon cancer cell migration [74]. Furthermore, the galectin-3-EGFR complex promotes lung cancer stemness by activating downstream signaling and upregulating Sox-2 expression via c-Myc [75]. Binding of galectin-3 to MUC1, a highly glycosylated transmembrane mucin protein, enhances MUC1 interaction with EGFR, thereby facilitating EGFR dimerization and activation [76]. Additionally, PDGFR β has been identified as an interactor for galectin-3 and may activate the downstream signaling [77]. Extracellular galectin-3 can act as the glycan bridge between the host and pathogens or binding partners for viral infection [78, 79]. Galectin-3 can interact with herpes simplex virus type 1 to facilitate the attachment of HSV-1 to host cells, which is blocked when transmembrane mucins bind to galectin-3 [14]. Exosomes derived from HIV-infected dendritic cells can transmit HIV-1 infection through fibronectin and galectin-3 and induce robust viral replication [80]. In the present study, we show that exogenously added galectin-3 proteins enhance influenza virus infection at the viral adsorption step (Fig. 9e and f). It is presumed that extracellular galectin-3 may bind to RTKs on the cell surfaces to assist the process of influenza virus infection through activation of the downstream signaling. Extracellular galectin-3 may also promote nuclear import and/or export of the vRNP (Fig S4), resulting in enhancing viral infection. More detailed study is needed to clarify which step(s) of viral life cycle is influenced by extracellular galectin-3 protein. Moreover, galectin-3 exhibits pleiotropic biological functions [81] and plays a dual role in virus infection [78]. It is possible that galectin-3 affects influenza virus infection through different strategies not directly on virus replication process. Since our treatment regimen is not optimized, we cannot exclude potential effects of extracellular galectin-3 on different stages of viral life cycle. Therefore, the roles and detailed mechanisms of action of extracellular galectin-3 in influenza virus infection are worthy of further exploration.

The galectin-3 gene consists of six exons and five introns and encodes a 29–35 kDa protein [82]. Galectin-3 is composed of an amino terminal half containing Gly-X-Tyr tandem repeats and a carboxyl terminal half containing the CRD with high affinity to *N*-acetyllactosamine [83]. The unique N-terminal domain (NTD) is required for multimerization of galectin-3 and intracellular protein-protein interaction [46, 84]. The NTD has an N-terminal region with a phosphorylation site at serine 6 [85] and a collagen-α-like sequence cleavable by matrix metalloproteases [86]. Extracellular galectin-3 can bind to glycans on cell surface glycoproteins or glycolipids through its CRD, which helps cell–cell or cell–extracellular matrix interaction and cell signaling transduction [81]. Galectin-3 also binds to intracellular proteins with its NTD or CRD, and such interaction may be independent of carbohydrate recognition. In the cytoplasm, Bcl-2 is the first molecule to be identified as a galectin-3-binding ligand. The association between galectin-3 and Bcl-2 is CRD-dependent because competition with lactose abolishes their association [87]. Furthermore, galectin-3 positively regulates Notch 1 signaling pathway in ovarian cancer cells and directly interacts with Notch 1 intracellular domain through its CRD [88]. Moreover, galectin-3 can bind directly to Gemin4, one component of nuclear complexes containing survival of motor neuron (SMN) protein, by both CRD and NTD and facilitate splicing process [89]. Galectin-3 is also involved in the Wnt/β-catenin signaling pathway and interacts with β-catenin through its CRD in a carbohydrate-dependent manner [90]. However, β-catenin is not a glycoprotein. This study suggests that sugar existence changes the conformation of galectin-3 and results in disrupting the binding of galectin-3 to β-catenin. Moreover, in HIV-1 infection, galectin-3 interacts with Alix, which is known to coordinate with the endosomal sorting complex required for transport (ESCRT), through its NTD that stabilizes the Alix-Gag p6 complex and promotes HIV-1 budding [12]. In the current study, we demonstrate that galectin-3 is associated with viral NS1 and NP proteins (Fig. 8). Galectin-3 can interact with hnRNP A2/B1 [47], which has been identified to bind both viral NS1 and NP proteins [50, 51]. It is possible that hnRNP A2/B1 is directly or indirectly involved in the association between galectin-3 and the viral NS1 or NP through forming a complex. So far, no study has indicated that NP and NS1 proteins are glycoproteins, thus excluding the possibility that galectin-3 binds to the NP or NS1 protein via its CRD. Therefore, we speculate that galectin-3 may bind to the viral NP or NS1 by its NTD. The interactions and functions of galectin-3 with intracellular proteins are complicated. Our results also raise questions about whether sugar is required for the binding of galectin-3 to various viral proteins and which domain of galectin-3 is critical for this binding. These issues warrant further investigation.

Galectin-3 can exist as a monomeric protein at low concentrations and forms homodimers at high concentrations [91]. In the present study, immunoblot analysis of total cell lysates from A549 cells with the anti-galectin-3 antibody shows a single band at 30 kDa (Figs. 3a and 4b and d). Lentivirus-mediated shRNA knockdown of galectin-3 in A549 cells abrogated to a large extent not only intracellular, but also extracellular galectin-3 expression. In galectin-3-knockdown A549 clones, hardly any bands were detectable in immunoblots with the anti-galectin-3 antibody. Regarding mouse samples, one major band at 30 kDa and another faint band at ~ 35 kDa were detected in mouse BAL fluid and lung tissue (Fig. 1b, Additional file 1: Fig. S1a). We used a rabbit polyclonal antibody (H-160) raised against amino acids 1-160 mapping at the N-terminus of human galectin-3, which was reactive to human, mouse, and rat galectin-3 due to high amino acid sequence homology (~ 80%) among the three species. In human umbilical vein endothelial cells, galectin-3 displays one major band at 25 kDa and another faint band at 35 kDa detected by immunoblotting [92]. In canine MDCK and human promonocytic HL-CZ cells, two clear bands at approximately 30 kDa were also detected by immunoblotting [19]. Galectin-3 monomers, dimers, and higher order oligomers may exist, and secreted galectin-3 may also undergo proteolytic processing in the NTD by matrix metalloproteinases. It is conceivable that different isoforms or cleaved products with various sizes may be present in different cell or tissue types. Therefore, we presume that the extra band of galectin-3 around ~ 35 kDa in immunoblots that we detected in mouse samples appeared to be a galectin-3 isoform, rather than other galectins, such as galectin-1, galectin-8, and galectin-9, present in the airway cross-reactive with the anti-galectin-3 antibody.

Development of novel therapeutic strategies by targeting influenza polymerase complex has been explored. Apart from being essential for the biological processes of viral transcription and replication, the RdRp also determines viral pathogenicity and host adaptation [93, 94]. Furthermore, several unique enzymatic properties and highly conserved structures among influenza A, B, and C strains make the RdRp an attractive target for the development of new antiviral drugs. Baloxavir marboxil, an anti-influenza drug approved by the US Food and Drug Administration (FDA) in 2018, inhibits cap-dependent endonuclease of the PA subunit and is effective against influenza A and B viruses [95]. In addition, numerous small molecule inhibitors have been identified to disrupt the PA activity [96, 97]. Other strategies to inhibit viral RdRp activity is to interfere with proper assembly of the polymerase complex through disrupting protein–protein interactions, such as the PA–PB1 interaction [98, 99]. In the present study, we show that galectin-3 interacts with the PA subunit and enhances the PA–PB1 interaction. However, whether such effects impact the endonuclease activity of the viral PA requires further investigation. More importantly, our findings identify galectin-3 as a potential target for developing anti-influenza drugs.

Identification of galectin-3 that contributes to the pathogenesis of IAV adds to our understating of the roles of host factors in participating in influenza virus infection. Our results show that upregulation of galectin-3 in IAV-infected cells facilitates nuclear import of the vRNP and subsequent viral RNA synthesis, which may also result in increased nuclear export of the vRNP at the late stage of the viral life cycle. However, the impact of galectin-3 on nuclear export of the vRNP requires detailed investigation. Furthermore, galectin-3 promotes the transcription and replication of IAV through interacting with the PA subunit of the RdRp, thereby enhancing virus production. It has been documented that influenza viral proteins interact with numerous host proteins, some of which can help viral replication [100]. In the present study, apart from its intracellular functions, extracellular galectin-3 can also enhance influenza virus infection. However, extracellular roles of galectin-3 in influenza virus infection merit further studies. We report that influenza virus can take advantages of galectin-3 to enhance replication of influenza virus. Furthermore, our findings provide potential therapeutic interventions and suggest galectin-3 as a potential therapeutic target for influenza.

## Conclusion

IAV requires host factors to support its replication and transcription. Therefore, studies on virus–host interactions would help us understand the viral life cycle and discover novel strategies against influenza. Here, we show that galectin-3, a β-galactoside-binding animal lectin, is upregulated in the airway during influenza virus infection and contributes to viral pathogenesis. Mice lacking galectin-3 are resistant to IAV infection. Furthermore, galectin-3 is associated with the PA subunit of the RdRp complex in the infected lung epithelial cells and facilitates viral RNA synthesis. Our findings identify galectin-3 as a novel target for the development of anti-influenza drugs.

## Supplementary Information

**Additional file1.** Additional figures. **Fig. S1** Galectin-3 is upregulated in the BAL fluid of mice following IAV infection. Mice were intratracheally inoculated with IAV (10^5^ PFU) at day 0, and the BAL fluid was collected at different time points. **a** Immunoblotting of galectin-3 in the BAL fluid of individual mice. **b** The intensity of the 30-kDa band corresponding to galectin-3 was determined by densitometric analysis, and relative expression levels of galectin-3 at different time points after viral infection were compared, where the ratio of day 0 was arbitrarily set to 1. Values shown are mean ± SD (n = 5). **Fig. S2** Recombinant mouse galectin-3 proteins induce macrophage migration at low concentration as well as binds to IAV and inhibits viral hemagglutination activity at high concentrations. **a, b** Microscopic images (**a**) and quantification of migratory cells (**b**) in the Boyden chamber assay. RAW 264.7 cells and various concentrations of recombinant galectin-3 proteins were applied to the upper and lower chambers, respectively. After 6 h, cells that migrated through the membrane to the lower surface were stained and quantified. The number of migratory cells was the average of the cells counted in three randomly selected fields in each well (n = 3). **c** Binding of galectins to IAV. Serial two-fold dilutions of galectin-3 or galectin-1, ranging from 100 μg/well to 6.25 μg/well, were applied to 96-well plates coated with IAV (4 HAU/well). The bound galectin-3 and galectin-1 proteins were detected by ELISA with anti-galectin-3 and anti-galectin-1 antibodies, respectively. Note that galectin-3 bound to IAV much weaker than galectin-1. **d** Hemagglutination inhibition activity of galectin-3. IAV (2 HAU) was incubated with various concentrations of galectin-3 for 60 min, followed by addition of human erythrocyte suspension. After an additional 60-min incubation, the presence or inhibition of hemagglutination was recorded. **Fig. S3** Treatment with recombinant mouse galectin-3 proteins does not impact body weight and survival of IAV-infected mice. Groups of C57BL/6 mice were intrantracheally inoculated with IAV (10^6^ PFU) at day 0 and treated with mouse galectin-3 proteins (50 μg) or BSA at days 2, 4, and 5 p.i. via the same route. **a** Changes in body weights from day 0 through day 4 while all mice were still alive. Body weights were recorded and expressed as the percentage of pre-infection (day 0) body weight. **b** Kaplan-Meier survival curves. Values shown are mean ± SD (n = 5). **Fig. S4** Exogenous galectin-3 affects the nucleocytoplasmic distribution of the vRNP complex in IAV-infected A549 cells. **a, b** A549 cells treated with recombinant human galectin-3 proteins (2 μg/ml) or untreated cells were infected with IAV at an MOI of 5 for 60 min, fixed, and then permeabilized at 2 h, 3.5 h, 4.5 h, and 6 h p.i. for immunofluorescence staining with the anti-NP antibody. Nuclei were stained with DAPI. Localization of the viral NP indicative of the vRNP complex (**a**) and quantitative analysis of nucleocytoplasmic distribution of the NP at 6 h p.i. (n = 4) (**b,** **c**). NP-positive (green staining) cells were examined for their nuclear/cytoplasmic localization at 6 h p.i. Representative images (original magnification × 200, scale bar = 50 μm) (**a**) and percentages of the NP observed only in the nucleus (**b**) and in both the nucleus and cytoplasm (**c**) are shown.

## Data Availability

All data generated in this study are available from corresponding author on reasonable request.

## Abbreviations

AEC: 3-Amino-9-ethylcarbazole
Alix: ALG-2-interacting protein X
BAL: Bronchoalveolar lavage
BMDM: Bone marrow-derived macrophages
BSA: Bovine serum albumin
cRNA: Complementary RNA
CRD: Carbohydrate-recognition domain
DMEM: Dulbecco’s modified Eagle’s medium
EGFR: Epidermal growth factor receptor
ELISA: Enzyme-linked immunosorbent assay
ESCRT: Endosomal sorting complex required for transport
HA: Hemagglutinin
HAU: Hemagglutinating unit
hnRNP: Heterogeneous nuclear ribonucleoprotein
HRP: Horseradish peroxidase
IAV: Influenza A virus
LD_50_: Median lethal dose
M1: Matrix protein 1
MOI: Multiplicity of infection
NLRP3: The nucleotide oligomerization domain-like receptor protein 3
NA: Neuraminidase
NP: Nucleoprotein
NTD: N-terminal domain
NS1: Non-structural protein 1
PA: Polymerase acidic protein
PB1: Polymerase basic protein 1
PB2: Polymerase basic protein 1
PBS: Phosphate-buffered saline
PCR: Polymerase chain reaction
PDGFR: Platelet-derived growth factor receptor
PFU: Plaque-forming unit
p.i.: Post-infection
RT-qPCR: Reverse transcription quantitative real-time polymerase chain reaction
RTKs: Receptor tyrosine kinases
RdRp: RNA-dependent RNA polymerase
shRNA: Small hairpin RNA
vRNA: Viral RNA
vRNP: Viral ribonucleoprotein
snRNP: Small nuclear ribonucleoprotein
SD: Standard deviation
SMN: Survival of motor neuron
WT: Wild-type
VEGFR: Vascular endothelial growth factor receptor
