# Supplementary material for: Upregulation of galectin-3 in influenza A virus infection promotes viral RNA synthesis through its association with viral PA protein
Source: J Biomed Sci. 2023 Feb 23;30:14. doi: 10.1186/s12929-023-00901-x (PMC9948428; doi:10.1186/s12929-023-00901-x)
Supplement: Supplementary file 1 — Additional file1. Additional figures. Fig. S1 Galectin-3 is upregulated in the BAL fluid of mice following IAV infection. Mice were intratracheally inoculated with IAV (105 PFU) at day 0, and the BAL fluid was collected at different time points. a Immunoblotting of galectin-3 in the BAL fluid of individual mice. b The intensity of the 30-kDa band corresponding to galectin-3 was determined by densitometric analysis, and relative expression levels of galectin-3 at different time points after viral infection were compared, where the ratio of day 0 was arbitrarily set to 1. Values shown are mean ± SD (n = 5). Fig. S2 Recombinant mouse galectin-3 proteins induce macrophage migration at low concentration as well as binds to IAV and inhibits viral hemagglutination activity at high concentrations. a, b Microscopic images (a) and quantification of migratory cells (b) in the Boyden chamber assay. RAW 264.7 cells and various concentrations of recombinant galectin-3 proteins were applied to the upper and lower chambers, respectively. After 6 h, cells that migrated through the membrane to the lower surface were stained and quantified. The number of migratory cells was the average of the cells counted in three randomly selected fields in each well (n = 3). c Binding of galectins to IAV. Serial two-fold dilutions of galectin-3 or galectin-1, ranging from 100 μg/well to 6.25 μg/well, were applied to 96-well plates coated with IAV (4 HAU/well). The bound galectin-3 and galectin-1 proteins were detected by ELISA with anti-galectin-3 and anti-galectin-1 antibodies, respectively. Note that galectin-3 bound to IAV much weaker than galectin-1. d Hemagglutination inhibition activity of galectin-3. IAV (2 HAU) was incubated with various concentrations of galectin-3 for 60 min, followed by addition of human erythrocyte suspension. After an additional 60-min incubation, the presence or inhibition of hemagglutination was recorded. Fig. S3 Treatment with recombinant mouse galectin-3 proteins d [file 12929_2023_901_MOESM1_ESM.docx]

**Additional file 1 for**

**Upregulation of galectin-3 in influenza A virus infection promotes viral RNA synthesis through its association with viral PA protein**

Mei-Lin Yang, Yi-Cheng Chen, Chung-Teng Wang, Hao-Earn Chong, Nai-Hui Chung, Chia-Hsing Leu, Fu-Tong Liu, Michael M. C. Lai, Pin Ling, Chao-Liang Wu and Ai-Li Shiau

**
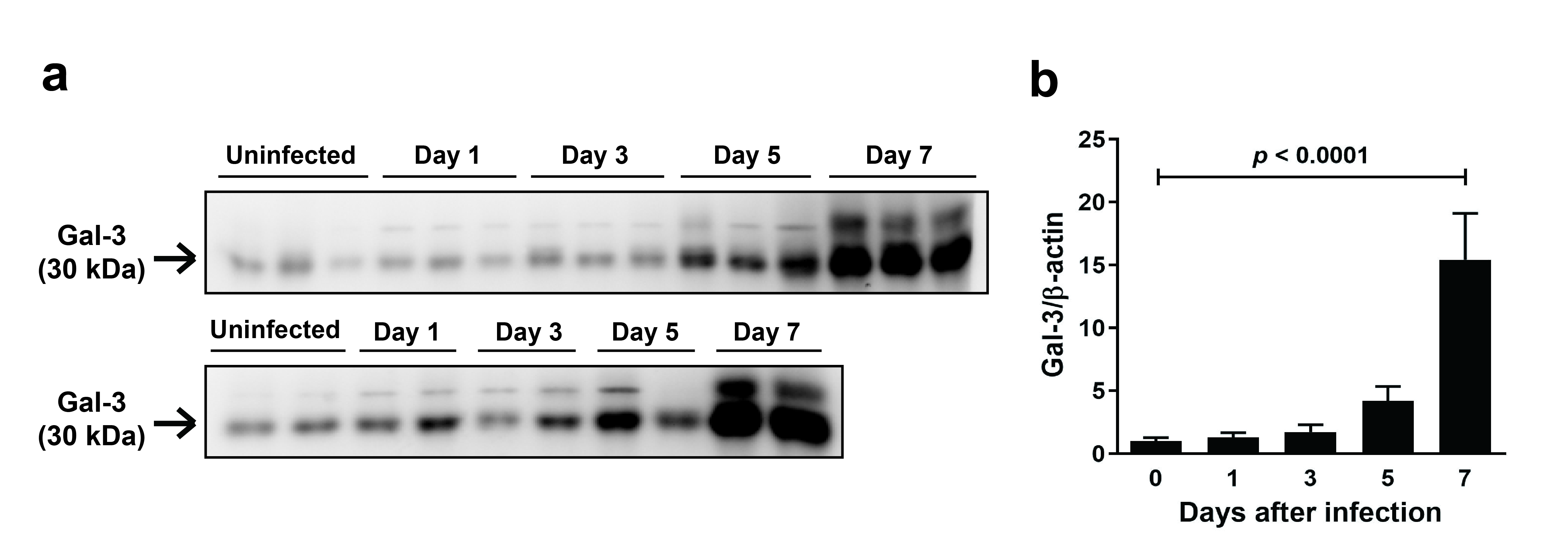
**

**Additional file 1: Fig. S1** Galectin-3 is upregulated in the BAL fluid of mice following IAV infection. Mice were intratracheally inoculated with IAV (10^5^ PFU) at day 0, and the BAL fluid was collected at different time points. **a** Immunoblotting of galectin-3 in the BAL fluid of individual mice. **b** The intensity of the 30-kDa band corresponding to galectin-3 was determined by densitometric analysis, and relative expression levels of galectin-3 at different time points after viral infection were compared, where the ratio of day 0 was arbitrarily set to 1. Values shown are mean ± SD (n = 5).


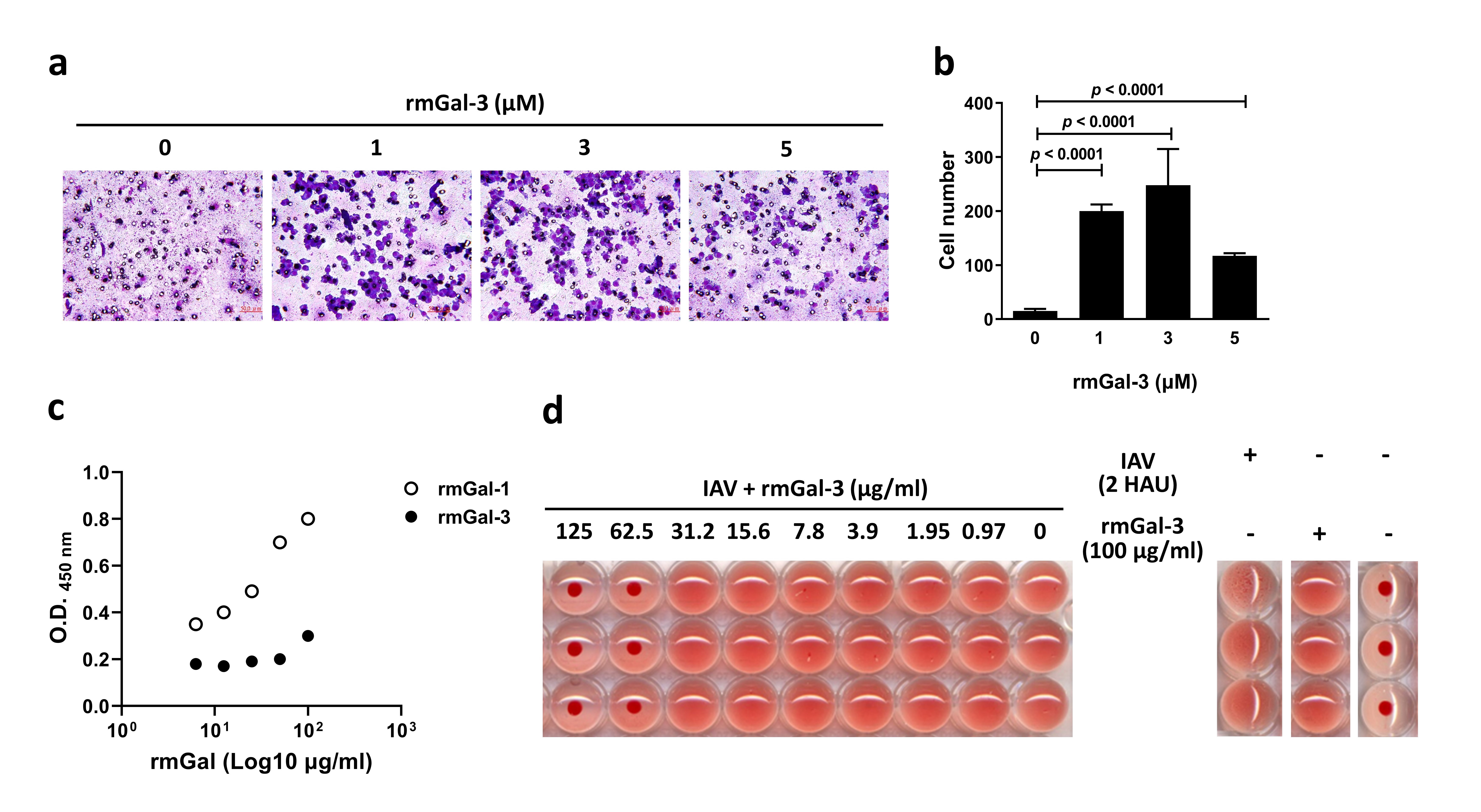
**Additional file 1: Fig. S2** Recombinant mouse galectin-3 proteins induce macrophage migration at low concentration as well as binds to IAV and inhibits viral hemagglutination activity at high concentrations. **a, b** Microscopic images **(a)** and quantification of migratory cells **(b)** in the Boyden chamber assay. RAW 264.7 cells and various concentrations of recombinant galectin-3 proteins were applied to the upper and lower chambers, respectively. After 6 h, cells that migrated through the membrane to the lower surface were stained and quantified. The number of migratory cells was the average of the cells counted in three randomly selected fields in each well (n = 3). **c** Binding of galectins to IAV. Serial two-fold dilutions of galectin-3 or galectin-1, ranging from 100 μg/well to 6.25 μg/well, were applied to 96-well plates coated with IAV (4 HAU/well). The bound galectin-3 and galectin-1 proteins were detected by ELISA with anti-galectin-3 and anti-galectin-1 antibodies, respectively. Note that galectin-3 bound to IAV much weaker than galectin-1. **d** Hemagglutination inhibition activity of galectin-3. IAV (2 HAU) was incubated with various concentrations of galectin-3 for 60 min, followed by addition of human erythrocyte suspension. After an additional 60-min incubation, the presence or inhibition of hemagglutination was recorded.

**
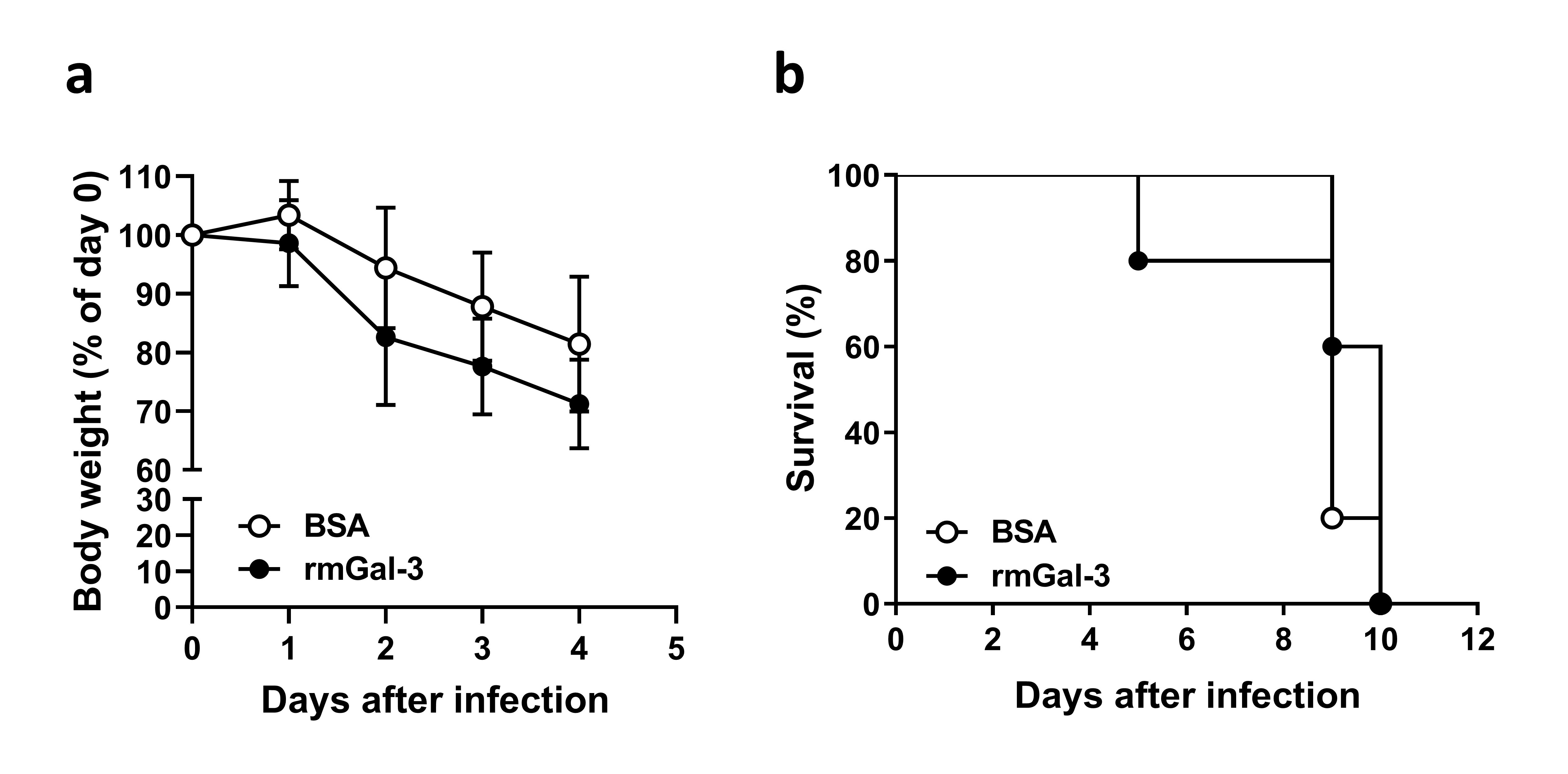
**

**Additional file 1: Fig. S3** Treatment with recombinant mouse galectin-3 proteins does not impact body weight and survival of IAV-infected mice. Groups of C57BL/6 mice were intrantracheally inoculated with IAV (10^6^ PFU) at day 0 and treated with mouse galectin-3 proteins (50 μg) or BSA at days 2, 4, and 5 p.i. via the same route. **a** Changes in body weights from day 0 through day 4 while all mice were still alive. Body weights were recorded and expressed as the percentage of pre-infection (day 0) body weight. **b** Kaplan-Meier survival curves. Values shown are mean ± SD (n = 5).

**
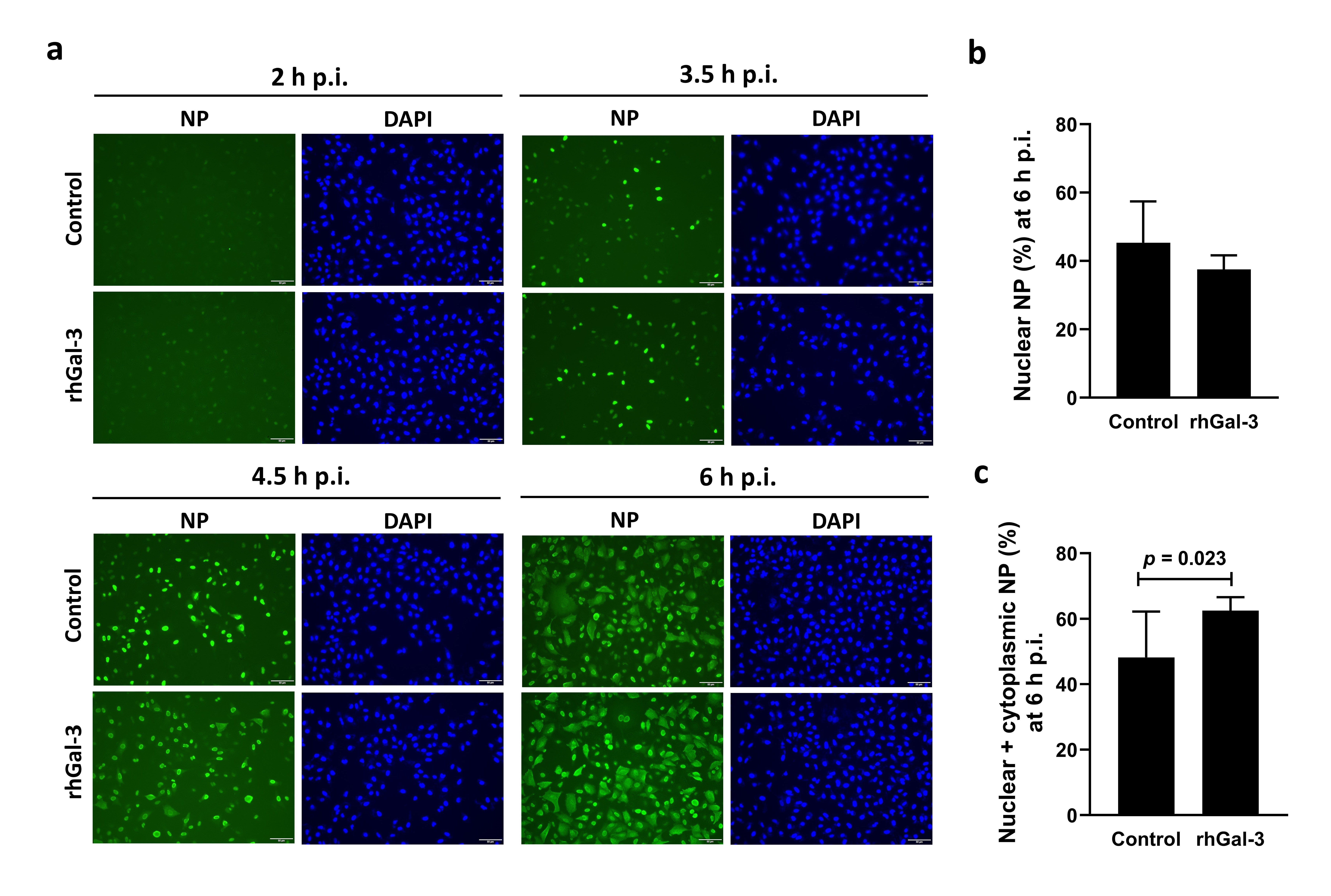
**

**Additional file 1: Fig. S4** Exogenous galectin-3 affects the nucleocytoplasmic distribution of the vRNP complex in IAV-infected A549 cells. **a, b** A549 cells treated with recombinant human galectin-3 proteins (2 μg/ml) or untreated cells were infected with IAV at an MOI of 5 for 60 min, fixed, and then permeabilized at 2 h, 3.5 h, 4.5 h, and 6 h p.i. for immunofluorescence staining with the anti-NP antibody. Nuclei were stained with DAPI. Localization of the viral NP indicative of the vRNP complex (**a**) and quantitative analysis of nucleocytoplasmic distribution of the NP at 6 h p.i. (n = 4) (**b, c**). NP-positive (green staining) cells were examined for their nuclear/cytoplasmic localization at 6 h p.i. Representative images (original magnification × 200, scale bar = 50 μm) (**a**) and percentages of the NP observed only in the nucleus (**b**) and in both the nucleus and cytoplasm (**c**) are shown.
